# Identification of stable and high-yielding maize genotypes using BLUP-based stability and multi-trait selection indices across diverse environments

**DOI:** 10.3389/fpls.2026.1858657

**Published:** 2026-07-21

**Authors:** Dharmendra Kumar, Prashant Bisen, Vikram Jeet Singh, Shridhar Ragi, Hitesh Kumar Yadav, Shekharappa Nandakumar, Sonu, Ankit Singh, Shivani Dubey, Vinay Kumar Chourasiya, Sudheer Kumar Pathak, Satyarth Gupta, Teepu Patel, Ashok Kumar, Shambhoo Prasad, Firoz Hossain, Subhash Chandra Vimal

**Affiliations:** 1 Acharya Narendra Deva University of Agriculture and Technology, Ayodhya, India; 2 India Council of Agricultural Research (ICAR)-Indian Agriculture Research Institute, New Delhi, India; 3 India Council of Agricultural Research (ICAR)-Central Arid Zone Research Institute, Regional Research Station (RRS), Jaisalmer, India; 4 New Mexico State University, Agricultural Science Center (ASC), Clovis, NM, United States; 5 Department of Genetics and Plant Breeding, School of Agriculture, Institute of Technology and Management University (ITMU), Gwalior, MP, India

**Keywords:** genetic gain, genotype × environment interaction, heritability, maize, multi-trait selection, phenotypic stability

## Abstract

Maize (*Zea mays* L.) is one of the most important cereal crops worldwide and serves as a major source of food, feed, and industrial raw material; however, identification of high-yielding and stable maize genotypes across diverse environments is a key objective of maize improvement programs. A total of 60 maize genotypes were evaluated across four environments [summer 2023 (E_1_), winter 2023 (E_2_), summer 2024 (E_3_), and winter 2024 (E_4_)] in an alpha lattice design with two replications, comprising six incomplete blocks with 10 entries per block. Subsequently, 15 morphological and yield-related traits were assessed to identify superior and stable maize genotypes across environments. A pooled analysis of variance revealed highly significant effects of genotypes, environments, and genotype × environment interaction for all traits. Broad-sense heritability was high for grain yield (92.40%), biological yield (97.10%), cob yield (92.60%), and plant height (99.20%), whereas anthesis silking interval showed low heritability (12.70%), indicating strong environmental influence. Grain yield exhibited strong positive genotypic correlations with cob yield, biological yield, plant height, kernels per ear, ear length, seed index, and ear diameter. Factor analysis under MGIDI identified three factors explaining 76.06% of total variation, representing yield components, partitioning efficiency, and ear morphology traits. Based on 15% selection intensity, nine genotypes were selected with predicted genetic gains ranging from −0.523% for anthesis–silking interval (indicating a desirable reduction in ASI) to 50.6% for grain yield. The highest coincidence index was observed between MGIDI and FAI-BLUP (66.67%). Genotypes G26 and G27 were consistently selected across all indices, indicating superior performance and stability. These genotypes are promising candidates for multi-location evaluation and can serve as potential parents in hybridization programs aimed at improving grain yield and adaptability in maize.

## 1 Introduction

Maize (*Zea mays* L.) is one of the most important cereal crops worldwide, with a global production of approximately 1,513 million tonnes (FAO, 2025). It serves as a major source of food, feed, and industrial raw material and plays a vital role in global food and nutritional security (Omar et al., 2022; Vieira et al., 2025; Mut et al., 2022; Vidadala et al., 2025). With the global population projected to exceed 9.7 billion by 2050, improving maize productivity and adaptability has become a major priority for crop improvement programs worldwide (Jat et al., 2025).

In India, maize is cultivated across both *kharif* and *rabi* seasons, covering diverse agro-climatic zones that vary considerably in temperature, photoperiod, and rainfall, resulting in substantial environmental variability that influences genotype performance and complicates the identification of stable and widely adapted cultivars (Directorate of Economics and Statistics, 2024). Despite its strategic importance, the rate of genetic improvement in maize grain yield has not always matched the increasing demand driven by expanding requirements for livestock feed, human consumption, and industrial utilization. In India, maize demand continues to rise due to increasing use in poultry feed and processing industries. However, further yield enhancement remains challenging because grain yield is a complex quantitative trait governed by the simultaneous expression of multiple morphological and physiological components, each influenced by genotype × environment interaction (GEI) (Singhal et al., 2022; Kumar et al., 2025). Traits such as days to anthesis, days to silking, anthesis–silking interval (ASI), plant height, ear length, ear diameter, number of kernels per ear, seed index, biological yield, cob yield, harvest index, and shelling percentage collectively determine the final grain yield of a genotype, and the improvement of any one trait in isolation is insufficient to guarantee meaningful yield progress (Yahaya et al., 2021; Adesoji et al., 2015). It is this multi-trait, multi-environment complexity that makes systematic and statistically rigorous genotype evaluation across diverse growing conditions both scientifically necessary and practically challenging.

Understanding the magnitude of GEI and its differential expression across traits is therefore a prerequisite for designing effective selection strategies in maize breeding programs. Several statistical approaches have been developed over the years to characterize GEI and identify stable, high-performing genotypes in multi-environment trials (METs). Classical stability models such as Wricke’s ecovalence (Wricke, 1962), Finlay–Wilkinson regression (Finlay and Wilkinson, 1963), Eberhart–Russell stability parameters (Eberhart and Russell, 1966), AMMI (Gauch and Zobel, 1988), and GGE biplot (Yan et al., 2000) have been widely used to assess phenotypic stability in maize and other cereal crops (Shirzad et al., 2025; Fauzan et al., 2025). The AMMI model combines analysis of variance (ANOVA) for additive main effects (genotype and environment) with principal component analysis through singular value decomposition to partition genotype × environment interaction (GEI) into meaningful interaction components, while the genotype plus genotype × environment interaction (GGE) biplot simultaneously visualizes genotype main effects and GEI, facilitating the identification of mega-environments and superior genotypes within specific environments (Shirzad et al., 2025; Yan and Kang, 2003). However, these classical approaches evaluate genotypes on the basis of individual traits, typically grain yield, and do not account for the simultaneous performance of multiple agronomically important traits that collectively determine the commercial and adaptive value of a genotype. Moreover, stability assessed in isolation from mean performance can mislead selection toward genotypes that are consistently mediocre rather than consistently superior (Olivoto et al., 2019). These limitations have driven the development of more comprehensive, multi-trait approaches that integrate mean performance and phenotypic stability into a unified selection framework.

In recent years, mixed-model-based approaches utilizing best linear unbiased prediction (BLUP) have gained wide acceptance in plant breeding for their ability to accurately estimate genotypic values by accounting for GEI and shrinking extreme estimates toward the population mean in proportion to their reliability (Mrode, 2014; Piepho et al., 2008). The weighted averages of absolute scores from BLUP interaction effects (WAASB) index and its integrated form WAASBY, which combines phenotypic stability and mean performance into a single parameter, have been proposed as more robust alternatives to classical stability indices for identifying genotypes that are both high-yielding and broadly adapted across environments (Olivoto et al., 2019; Yue et al., 2022b). Building on this framework, multi-trait selection indices including the multi-trait genotype–ideotype distance index (MGIDI), the multi-trait stability index (MTSI), the multi-trait mean performance and stability index (MTMPS), and the multi-trait index based on factor analysis and genotype–ideotype distance (FAI-BLUP) have been developed to enable simultaneous selection for multiple traits with defined breeding objectives (Olivoto et al., 2019; Olivoto et al., 2021; Rocha et al., 2017). These indices use factor analysis to group correlated traits into independent factors, compute the distance of each genotype from a defined ideotype, and rank genotypes based on their proximity to the ideal multi-trait profile, thereby providing an objective and comprehensive basis for selection decisions in complex multi-environment breeding programs (Olivoto and Nardino, 2021). Although these approaches provide substantial advantages for genotype evaluation and selection, each method emphasizes different aspects of genotype performance and may differ in its treatment of mean performance, stability, genotype × environment interaction, and trait interrelationships—for example, WAASB primarily focuses on stability, whereas WAASBY integrates stability and productivity. Similarly, MGIDI, MTSI, MTMPS, and FAI-BLUP differ in their underlying assumptions and selection criteria for ideotype-based ranking. Therefore, the use of multiple complementary approaches may provide a more comprehensive and reliable framework for identifying superior genotypes than reliance on a single selection method.

Despite the widespread cultivation of maize across diverse agro-climatic zones in India and the growing availability of genetically diverse germplasm, systematic multi-season evaluation of maize genotypes using advanced BLUP-based multi-trait selection tools remains limited in the Indian context. Several studies on maize GEI in India have employed stability and multivariate approaches; however, many investigations have focused on specific environments, single-season evaluations, or a limited set of selection parameters (Choudhary et al., 2019; Kumar et al., 2025). In addition, estimation of genotypic variability parameters such as variance components and heritability is important for understanding the magnitude of genetic variation and the potential response to selection in breeding populations. Evaluating genotypes across contrasting summer and winter seasons enables the identification of genotypes with genuine broad adaptability as well as season-specific superior performers, thereby providing actionable information for varietal deployment across diverse maize-growing environments of northern India (Yue et al., 2022a; Nagesh et al., 2025). In view of the details above, the present study was undertaken with the following objectives: (i) to assess the nature and magnitude of genetic variability, heritability, and genotype × environment interaction for morphological and yield-related traits in 60 maize genotypes evaluated across four environments comprising two summer and two winter cropping seasons, (ii) to estimate variance components and genotypic correlations among traits using restricted maximum likelihood (REML) within a linear mixed-effects model framework, (iii) to evaluate phenotypic stability and mean performance of genotypes simultaneously using BLUP-based WAASB and WAASBY indices, (iv) to identify superior and stable maize genotypes using four complementary multi-trait selection indices such as MGIDI, MTSI, MTMPS, and FAI-BLUP, and (v) to compare the selection efficiency of these indices through coincidence index analysis and to identify consensus elite genotypes for advancement in the maize improvement program. The information generated from this study is expected to provide a sound scientific basis for the identification of high-yielding, broadly adapted maize genotypes and for the design of efficient crossing programs targeting simultaneous improvement of grain yield, yield-attributing traits, and phenotypic stability under the diverse growing environments of northern India.

## 2 Materials and methods

### 2.1 Plant materials and multi-season trials

The 59 genotypes along with one check (**Supplementary Table 1**) were evaluated across four environments (E), namely, summer 2023 (E_1_), winter 2023 (E_2_), summer 2024 (E_3_), and winter 2024 (E_4_), at Research Farm, Department of Genetics and Plant Breeding, Acharya Narendra Deva University of Agriculture and Technology, Kumarganj, Ayodhya (26°47′ N, 82°12′ E, 113 MSL). The check genotype was included as a reference cultivar to facilitate comparison of the performance of experimental entries across seasons and environments. The trial was laid out using an alpha lattice design with two replications, comprising six incomplete blocks with 10 entries per replication, to improve precision by controlling local field heterogeneity. The use of an alpha lattice design reduces residual variation and increases the efficiency of genotype comparisons when a relatively large number of entries are evaluated simultaneously. The four environments represented combinations of year and season, namely, summer 2023 (E_1_), winter 2023 (E_2_), summer 2024 (E_3_), and winter 2024 (E_4_), thereby capturing variation arising from both seasonal and year-specific environmental conditions. Environmental heterogeneity among the four environments is illustrated in **Supplementary Figure 1**. This experimental setup facilitated the identification of superior maize genotypes with broad adaptability across contrasting environments. Each genotype was planted in four rows in each replication with row length 4 m, row-to-row spacing 60 cm, plant-to-plant spacing 30 cm, gross plot size 9.6 m^2^, and net plot size 7.2 m^2^. The sowing was undertaken on February 5, 2023 in E_1_, November 10, 2023 in E_2_, February 8, 2024 in E_3_, and November 12, 2024 in E_4_.

### 2.2 Morphological characterization

Data were recorded on five randomly selected plants from each genotype for all the studied traits. The observations were grouped into phenological, morphological, yield component, and productivity-related traits for better organization.

Phenological traits: (i) days to 50% tasseling (DT), recorded as the number of days from sowing to when 50% of plants in a plot exhibited tassel emergence; (ii) days to 50% silking (DS), recorded as the number of days from sowing to when 50% of plants showed silk emergence; (iii) anthesis–silking interval (ASI), estimated as the difference between days to tasseling and days to silking; and (iv) days to maturity (M), recorded by visual observation at physiological maturity (IBPGR, 1991).

Morphological and yield component traits: (v) plant height (PH), measured from ground level to the tip of the tassel; (vi) ear length (EL), measured after removing the husk from tip to base; (vii) ear diameter (ED), measured at the middle of the dehusked cob; (viii) number of kernel rows per ear (KRE), recorded by counting the total number of kernel rows in each cob; (ix) number of kernels per ear (KPE), recorded as the total number of kernels per selected cob; and (x) seed index (SI), recorded as the weight of 100 seeds (IBPGR, 1991).

Productivity traits: (xi) biological yield (BY) per plant, recorded as total dry biomass and expressed in g/plant; (xii) cob yield (CY) per plant, recorded after drying cobs to constant weight; (xiii) grain yield (GY) per plant, recorded as the weight of shelled kernels from selected plants; (xiv) harvest index (HI), calculated as the ratio of economic yield to biological yield; and (xv) shelling percentage (SP), calculated as the ratio of grain yield to cob yield per plant (Donald, 1968; Yue et al., 2022b).

### 2.3 Statistical analysis

#### 2.3.1 REML-BLUP analysis

To account for the respective influences of season and year on genotype performance, a linear mixed-effects model was fitted for each trait using the lmer function from the lme4 package (Bates et al., 2015) (Equation 1):

(1) yijkn = µ + Gi + Mj + Yk + GMij +

GYik +MYjk + GMYijk +REP n (j:k)+ BLOCKm(l:j:k)+ϵijkn

where $y_{ijklm}$ is the observed phenotypic value of the i-th genotype in the m-th incomplete block nested within the l-th replication under the jth season and kth year; μ  is the overall mean; $G_{i}$, $M_{j}$, and $Y_{k}$ are the effects of genotype, season, and year, respectively; $GM_{ij}$, $GY_{ik}$, $MY_{jk}$, and $GMY_{ijk}$ represent their interaction effects; $REP_{l(j:k)}$ denotes the replication effect nested within season and year; $BLOCK_{m(l:j:k)}$ represents the incomplete block effect nested within replication, season, and year; and $\epsilon_{ijklm}$ is the residual error term.Variance components were first estimated through REML, and the estimated values were subsequently used for prediction of genotypic values using BLUP [Dempster et al., 1977). Therefore, these procedures do not represent independent analyses but rather sequential steps within a single integrated mixed-model framework. Since the present study employed a REML–BLUP mixed-model approach for multi-environment analysis, a separate Bartlett’s test for homogeneity of variance prior to combined analysis was not performed, as variance components were estimated directly within the model structure. Phenotypic variance (σ²_p_), genotypic variance (σ²g), and broad-sense heritability (h²bs) were estimated from the variance components obtained through the mixed model, with broad-sense heritability estimated as:


$$h_{bs}^{2}=\frac{\sigma_{g}^{2}}{\sigma_{p}^{2}}$$

The estimated variance components were subsequently utilized within the same mixed-model framework for prediction of genotypic values using Best Linear Unbiased Prediction (BLUP), stability assessment, and multi-trait selection. The mixed model can be represented in matrix notation as (Equation 2):

(2) Y=Xβ+Zu+ϵ

where Y is the vector of phenotypic observations; β is the vector of fixed effects (block means within environments); u is the vector of random genotype and genotype × environment interaction effects; X and Z are the incidence matrices relating observations to fixed and random effects, respectively; and ϵ is the vector of random residual errors.The variance components of agronomic traits were estimated by REML and subsequently used for BLUP prediction, genotype × environment interaction analysis, stability assessment, and multi-trait selection using the “metan” package (version 1.19.0) (Olivoto and Lúcio, 2020). In the present study, environments were defined as combinations of year and season (summer 2023, winter 2023, summer 2024, and winter 2024); therefore, year × season variation was implicitly captured within the environmental effect included in the model. Statistical analyses were performed using R statistical software (R Core Team, 2024).

#### 2.3.2 Evaluation of factor analytic covariance structure

A first-order factor analytic model (FA1) was fitted for grain yield using the sommer package in R. The FA1 model allows heterogeneous genetic variances across environments and accounts for genetic correlations among environments through a latent factor structure. Model performance was evaluated using restricted maximum likelihood (REML), and model fit was compared with the original genotype × environment interaction model using log-likelihood, Akaike Information Criterion (AIC), and Bayesian Information Criterion (BIC). The consistency of genotype predictions between the two models was assessed by Pearson’s correlation between predicted genotypic effects and comparison of genotype rankings.

### 2.4 Multi-trait-based stability evaluation methods

The BLUP-predicted genotypic values obtained from the mixed-model analysis were subsequently used as input for all multi-trait analyses, including MGIDI, MTSI, MTMPS, and FAI-BLUP (Olivoto and Lúcio, 2020). Prior to index computation, trait values were rescaled according to the breeding objective (higher or lower desired values) to standardize variables and facilitate comparisons among traits with different measurement scales (Olivoto and Lúcio, 2020). These methods were used to identify the ideotype or ideal type genotypes, considering all the studied traits. In the present investigation, only days to 50% flowering was desired for a lower mean value, and the rest of the yield traits were desired for a higher mean value.

#### 2.4.1 Multi-trait genotype-ideotype distance index

Multi-trait genotype-ideotype distance index (MGIDI) was computed in four steps using mgidi() function implemented in the “metan” R package, which is as follow: rescaling the traits, factor analysis, ideotype designing and calculating the MGIDI index by using the methodology of (Olivoto and Nardino, 2021) (Equation 3).

(3) $$MGIDI_{i}=\;\sqrt{\sum_{j=1}^{f}{\left(Y_{ij}-Yj\right)}^{2}}$$

Where, *MGIDI_i_* is the multi-trait genotype-ideotype distance index for *i*-th genotype, *Y_ij_* is the score of the *i*-th genotype in the *j*-th factor, *Y_j_* is the score of the ideotype for the *j*-th factor, and *f* is the number of retained factors. The strength and weakness of genotype were assessed by calculating the proportion of the MGIDI of the i-th genotype explained by the j-th factor (ωij) which involved four steps: rescaling of traits, factor analysis, ideotype design, and calculation of the MGIDI index (Equation 4):

(4) $$\text{ωij}=\frac{\sqrt{D_{ij}^{2}}}{\sum_{j=1}^{f}\sqrt{D_{ij}^{2}}}$$

where, *D_ij_* is the distance between the *i*-th genotype and ideal genotype for the *j*-th factor. A trait with low contribution indicates that the genotypes within such trait are close to ideal genotype.

#### 2.4.2 Multi-trait stability index

The multi-trait stability index (MTSI) differs from the MGIDI in that it incorporates mean performance and stability values in the Fgp matrix through factor analysis, whereas MGIDI incorporates only BLUP mean. The genotype ranking was determined by calculating the Euclidean distance using the scores of each genotype compared to the score of the ideotype. MTSI was calculated by using the formula (Olivoto and Nardino, 2021) (Equation 5).

(5) $$MTSI_{i}=\sqrt{\sum_{j=1}^{f}{\left(F_{ij}-F_{j}\right)}^{2}}$$

Where, the MTSIi is the multi-trait stability index for the i-th genotype, Fij = j-th score of the i-th genotype; Fj = j-th score of the ideotype. MTSI utilizes the harmony between average performance and stability to identify genotypes that exhibit both high performance and stability.

#### 2.4.3 Multi-trait mean performance and stability index

The multi-trait mean performance and stability index (MTMPS) was computed using the “metan” R package. MTMPS differs from MTSI in that stability was assessed using Wricke’s ecovalence (Wi), whereas MTSI incorporates stability through the WAASB index (Equations 6, 7).

(6) X=μ+Lf+ϵ

Where X is a p×1 vector of rescaled observations; µ is a p×1 vector of standardized means; L is a p×*f* matrix of factorial loadings; f is a p×1 vector of common factors; and ϵ is a p×1 vector of residuals, being p and *f* the number of traits and common factors retained, respectively.

(7) $$MTMPS_{i}=\;\sqrt{a^{2}\sum_{j=1}^{f}{\left(F_{ij}-F_{j}\right)}^{2}}$$

Where MTMPSi is the multi-trait mean performance and stability index of the i-th genotype; Fij represents the j-th factor score of the i-th genotype, and Fj represents the score of the ideotype for the j-th factor.

#### 2.4.4 Multi-trait index based on factor analysis and genotype–ideotype–distance

Factor analysis and genotype-ideotype-distance (FAI-BLUP) calculates the spatial probability of each genotype by estimating its distance from the ideotype. This ranking will aid in the evaluation of the genotypes. The calculation formula of multi-trait index based on FAI-BLUP is as follow (Equation 8).

(8) $$P_{ij}=[\frac{\left(\frac{1}{d_{ij}}\right)}{\sum_{i=1;j=1}^{i=n;j=m}(\frac{1}{d_{ij}})}]$$

where *P_ij_* represents the probability that the *i*-th genotype is similar to the *j*-th genotype, *d_ij_* represents the genotype–ideotype distance from the *i*-th genotype to the *j*-th ideotype, *n* represents the total number of genotypes, and *m* represents the total number of ideotypes.

#### 2.4.5 Selection differential and genetic gain under selection

The genotype was selected under different growing environments through MGIDI values by considering a selection intensity of approximately 15%, and the selection differential in the percentage of population mean (ΔS%) was then computed for each trait as follows (Equation 9):

(9) $$\Delta S\%=\frac{\left(X_{s}\;-\;X_{0}\right)}{X_{0}}\times 100$$

Genetic gain percentage under selection (Δ*G*%) is calculated as follows (Equation 10):

(10) $$\Delta G\%=\frac{h^{2}(X_{s\;}-\;X_{0})}{X_{0}}\times 100$$

where *X_o_* is the mean for WAASBY index of the original population, *X_s_* is the mean for WAASBY index of the selected genotypes, *h*^2^ is the broad-sense heritability estimate for the respective trait, Δ*S* is the selection differential, and Δ*G* is the predicted genetic gain under selection. This rescaling facilitates the definition of an ideotype, originally proposed by Donald (1968), enabling the selection of genotypes closest to the ideal multi-trait profile.

#### 2.4.6 Figure generation and software used

All statistical analyses were performed using R software version 4.5.1 (R Core Team, 2025). Mixed-model analyses, variance component estimation, best linear unbiased prediction (BLUP), stability analyses, and multi-trait selection indices were performed using the metan package (Olivoto and Lúcio, 2020). Factor analytic covariance structure analyses were conducted using the sommer package (Covarrubias-Pazaran, 2016). Data manipulation and processing were performed using the dplyr package (Wickham et al., 2023) and tidyr package (Wickham and Girlich, 2024). Graphical visualizations were generated using ggplot2 (Wickham, 2016), GGally (Schloerke et al., 2024), circlize (Gu et al., 2014), and ggvenn (Yan, 2024). Final figures were exported in high-resolution format and arranged for publication.

## 3 Results

### 3.1 Mean performance and analysis of variance

**Figure 1:**
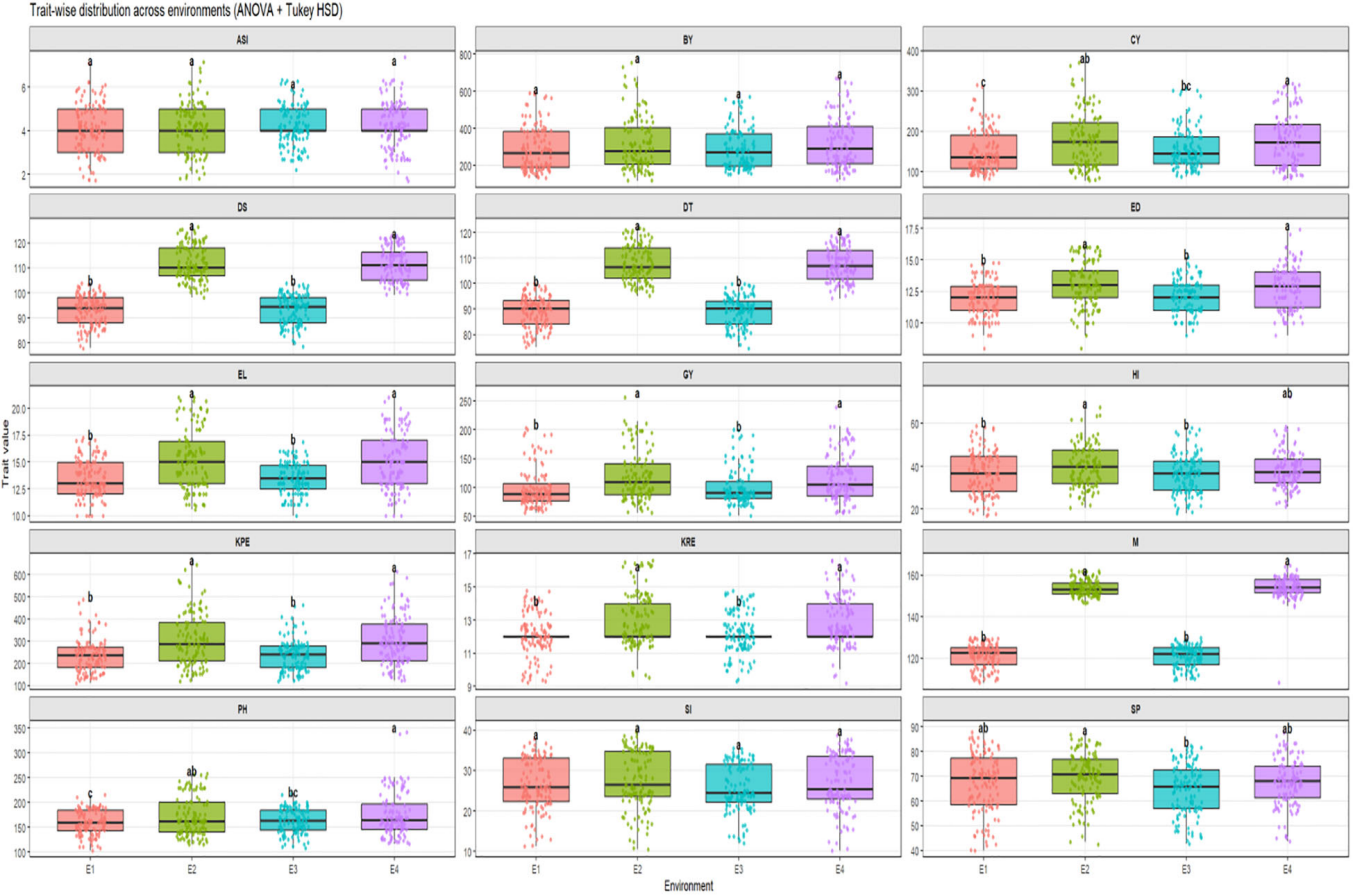
Boxplots depicting the distribution of genotype mean performance for yield and related traits evaluated across four environments (E1, E2, E3 and E4). Different lowercase letters indicate significant differences among environments for each trait according to Tukey's Honestly Significant Difference (HSD) test at P < 0.05.

Pooled analysis of variance (ANOVA) showed highly significant differences for maize genotypes, with a significant effect of GEI for all the studied traits, indicating substantial phenotypic variation among the 60 maize genotypes including check across the four test environments. The least significant difference (LSD) at the 5% probability level (*P* = 0.05) ranged from 0.84 for SI to 180.80 for KPE, indicating the predominance of genotype × environment interaction effects (**Table 1**). The days to 50% tasseling ranged from 87.71 to 105.19 days, with a mean value of 97.68 days, while days to 50% silking ranged from 91.35 to 109.55 days, with a mean of 101.90 days. The minimal ASI ranged from 2.00 to 7.00 days, with a mean value of 4.02 days, reflecting a vast variation in maturity from 127.62 to 142.37 days, with a mean of 136.32 days. The PH varied widely from 103.00 to 256.25 cm, with a mean value of 173.75 cm. The EL varied from 10.00 to 21.00 cm, while ED ranged from 8.00 to 17.00 cm, with a mean of 12.45 cm among the maize genotypes (**Figure 1**). The KRE ranged from 10.00 to 16.00, while KPE showed a vast variation from 112.00 to 645, with a mean value of 297.55. The SI varied from 10.25 to 39.43 g, with a mean of 27.48 g. The BY showed a wide variation from 118.00 to 754.00 g, with a mean value of 291.95 g, while the CY ranged from 77.00 to 370.00 g, with a mean value of 165.21 g. The GY likewise showed variation from 51.00 to 256.00 g, with a mean value of 112.23 g. The HI ranged from 17.04 to 63.40, with a mean value of 41.05, and SP ranged from 40.00 to 87.76%, with a mean value of 69.01%. The coefficient of variation (CV, %) was low to moderate for traits such as DS (1.15), DT (1.23), SI (1.61), CY (1.73), EL (3.27), SP (3.67), ED (3.86), PH (4.07), M (4.40), BY (4.59), GY (6.24), KRE (6.19), and HI (6.11). In contrast, higher CV values were recorded for KPE (33.32) and ASI (21.19) (**Table 1**).

**Table 1:** Pooled analysis of variance with mean sum of squares for yield and yield-attributing traits in four environments.

Source	DT	DS	ASI	M	PH	EL	ED	KRE	KPE	SI	BY	CY	GY	HI	SP
ENV	14059.50***	14076.62***	0.15*	42856.06***	5849.85***	106.49**	34.30**	34.67	244242.82***	72.41	36540.22***	22619.03***	14197.67***	360.25***	501.40**
BLOCK (REP * ENV)	1.75***	4.08**	1.12	22.58***	359.05***	3.72	7.24	0.552	7523.70***	0.41	714.76***	133.63***	12.13***	23.01***	5.08***
GEN	137.69***	141.54***	1.68	117.50**	8043.54**	29.55	14.15	8.82	70707.88***	336.37	129536.72***	27568.08***	10419.94***	597.51**	692.96**
GEN * ENV	62.07***	61.60***	1.26	76.64***	388.98***	3.15	1.31	1.291	10857.92***	2.91	1208.43***	671.75***	316.47***	36.20***	53.63**
Residuals	1.45	1.37	0.79	36.43	46.47	0.21	0.23	0.61	8422.57	0.18	195.63	8.14	46.02	5.34	6.09
CV (%)	1.23	1.15	21.19	4.40	4.07	3.27	3.86	6.19	33.32	1.61	4.59	1.73	6.24	6.11	3.67
LSD	2.37	2.31	1.75	11.89	13.43	0.92	0.94	1.53	180.80	0.84	27.55	5.62	13.36	4.55	4.86

DT, days to 50% tasseling; DS, days to 50% silking; ASI, anthesis silking interval; M, days to maturity; PH, plant height (cm); EL, ear length (cm); ED, ear diameter (cm); KRE, number of kernel rows per ear; KPE, number of kernels per ear; SI, seed index; BY, biological yield per plant (g); CY, cob yield per plant (g); GY, grain yield per plant (g); HI, harvest index; SP, shelling percentage; CV, coefficient of variation (%); LSD, least significant difference.

*p < 0.05; **p < 0.01; ***p < 0.001.

### 3.2 Variance components and likelihood ratio test

The likelihood ratio tests showed significant genotypic effects for all traits, indicating substantial genetic variability among the maize genotypes. The genotype × environment interaction was highly significant for most traits; however, ASI showed a non-significant GEI effect, indicating relatively limited environmental influence on genotype ranking for this trait. Phenotypic variance (*σ*^2p^) was highest for BY (16937.00), followed by KPE (9663.00), CY (3510.00), GY (1415.00), PH (1031.00), and SP (108.00), whereas genotypic variance (*σ*^2g^) was highest for BY (16100.44), followed by KPE (7838.29), CY (3388.54), GY (1267.65), PH (970.71), and SP (82.70). The broad-sense heritability (*h*^2bs^) estimates were high for PH (99.20%), BY (97.10%), CY (92.60%), GY (92.40%), HI (88.00%), and KPE (79.00%), moderate to high for ED (69.60%), EL (68.60%), KRE (63.10%), and SI (45.00%), and low for ASI (12.70%) (**Table 2**).

**Table 2:** Mean performance, likelihood ratio test, genetic parameters, and variance components for 15 quantitative traits evaluated in 60 maize genotypes across four environments.

Parameters	ASI	PH	EL	ED	KRE	KPE	SI	BY	CY	GY	HI	SP
Mean	4.02	173.75	14.28	12.45	12.70	297.55	27.48	291.95	165.21	112.23	41.05	69.01
SE	0.06	2.04	0.14	0.10	0.08	6.42	7.88	7.12	3.28	2.16	0.65	0.59
SD	1.09	35.04	2.37	1.67	1.45	110.23	135.83	122.25	56.25	37.17	11.20	10.05
Min	2.00 (C1 in E1)	103 (G1 in E1)	10.00 (G2 in E1)	8.00 (G15 in E1)	10.00 (G2 in E1)	112.00 (G12 in E1)	10.25 (G13 in E4)	118.00 (G17 in E2)	77.00 (G4 in E2)	51.00 (G42 in E3)	17.04 (G35 in E1)	40.00 (G36 in E1)
Max	7 (G17 in E1)	256.25 (G26 in E2)	21.00 (G22 in E2)	17.00 (G32 in E4)	16.00 (G13 in E2)	645 (G32 in E2)	39.43 (G30 in E2)	754.00 (G45 In E2)	370.00 (G45 in E2)	256.00 (G29 in E2)	63.4 (G2 in E2)	87.76 (G14 in E1)
Min ENV	E2 (3.89)	E1 (167.23)	E1 (13.58)	E1 (11.77)	E1, (12.27)	E1 (267.34)	E3 (26.70)	E1 (276.77)	E1 (152.20)	E1 (102.99)	E3 (39.84)	E3 (66.94)
Max ENV	E1, (4.14)	E4 (178.62)	E2 (14.99)	E2 (12.94)	E2 (13.08)	E2 (327.01)	E4 (59.16)	E2 (306.43)	E2 (175.28)	E2 (122.04)	E2 (42.62)	E2 (70.99)
Min GEN	G-30 (2.50)	G-1 (111.58)	G-21 (10.50)	G-15 (9.00)	G-9 (10.00)	G-12 (118.50)	G-13 (11.00)	G-17 (142.75)	G-1 (92.75)	G-17 (61.75)	G-54 (19.86)	G-36 (43.34)
Max GEN	G-14 (5.75)	G-32 (E4) (341.00)	G-26 (18.50)	G-26 (14.75)	G-13 (15.00)	G-32 (508.00)	G-57 (606.81)	G-45 (642.00)	G-45 (324.5)	G-59 (217.5)	G-10 (58.23)	G-3 (81.96)
*σ* ^2p^	1.17	1031.00	5.36	2.61	1.78	9663.00	22.0	16937	3510.00	1415.00	93.5	108
*σ* ^2g^	0.084	970.718	3.420	1.674	0.958	7838.292	41.819	16100.440	3388.544	1267.650	72.053	82.704
*h* ^2bs^	0.127	0.992	0.686	0.696	0.631	0.854	0.450	0.971	0.926	0.924	0.880	0.790
GEI*r*^2^	0.010	0.0156	0.259	0.142	0.0952	0.132	0.1000	0.0250	0.0585	0.0421	0.0001	0.165
AS	0.857	0.996	0.954	0.970	0.972	0.981	0.424	0.997	0.992	0.993	0.997	0.974
Rge	0.0124	0.200	0.823	0.468	0.258	0.909	0.100	0.860	0.795	0.555	0.0008	0.783
CVg	9.61	17.70	13.40	10.80	8.34	30.50	8.90	43.90	34.50	32.2	22.10	13.4
CVr	25.00	4.61	3.83	5.22	5.50	3.80	1.22	2.84	4.41	6.16	8.16	3.21
CV ratio	0.384	3.85	3.51	2.07	1.52	8.03	7.27	15.50	7.83	5.23	2.71	4.16
LRT_ge_	7.93	570.77	287.90	321.66	167.81	453.71	1192.44	1112.25	775.06	621.05	509.20	444.56
*P*-value	0.004	0.00003	0.00001	0.00006	0.00002	0.00001	0.00002	0.00007	0.00001	0.00004	0.00009	0.00001

Genotype (GEN) and genotype × environment interaction (G × E) effects were tested using likelihood ratio tests.

DT, days to 50% tasseling; DS, days to 50% silking; ASI, anthesis silking interval; M, days to maturity; PH, plant height (cm); EL, ear length (cm); ED, ear diameter (cm); KRE, number of kernel rows per ear; KPE, number of kernels per ear; SI, seed index; BY, biological yield per plant (g); CY, cob yield per plant (g); GY, grain yield per plant (g); HI, harvest index; SP, shelling percentage; SE, standard error; SD, standard deviation; CV, coefficient of variation; *σ*^2p^, phenotypic variance; *σ*^2g^, genotypic variance; *σ*^2ge^, GEI variance; *h*^2bs^, broad sense heritability (%); GEI*r*^2^, GEI coefficient of determination; AS, accuracy of genotype selection; Rge, association among genotypic values across environments; CVg, genotypic coefficient of variation; CVr, residual coefficient of variation; LRT_ge_, likelihood ratio test for GE interaction; *P*-value, probability value.

The proportion of variance due to the genotype × environment interaction (GEI*r*^2^) was highest for ear length (0.259), followed by HI (0.170), SP (0.165), ED (0.142), and KPE (0.132). Meanwhile, KRE (0.095), CY (0.058), GY (0.042), BY (0.025), PH (0.015), and ASI (0.010) exhibited relatively low GEI contributions. The selection accuracy (AS) value ranged from 0.42 for SI to 0.997 for HI and BY, with an accuracy of PH (0.996), GY (0.993), CY (0.992), KPE (0.981), SP (0.974), KRE (0.972), ED (0.970), EL (0.954), ASI (0.857), and SI (0.424). Genotypic correlation among the environments (rge) was high for KPE (0.909), BY (0.860), EL (0.823), CY (0.795), and SP (0.783), moderate for GY (0.555) and ED (0.468), and low for KRE (0.258), PH (0.200), SI (0.100), ASI (0.012), and HI (0.0008), indicating trait-specific consistency of maize genotypes performance across environments. The genetic coefficient of variation (CV_g_) ranged from 43.90% for BY to 8.34% for KRE, while the residual coefficient of variation (CV_r_) varied from 1.22% for SI to 25.00% for ASI. The CV ratio expanded unity for BY (15.50), KPE (8.03), CY (7.83), SI (7.27), and GY (5.23), indicating greater genetic variability for these traits than for the other traits. Likelihood ratio tests confirmed highly significant GEI for all the traits, with LRT_ge_ values that varied from 7.93 (ASI) to 1,192.44 (SI) (*p* < 0.001) (**Table 2**).

### 3.3 Evaluation of the FA1 covariance structure

A first-order factor analytic (FA1) covariance structure was fitted to evaluate whether modeling heterogeneous genetic variances and genetic correlations among environments improved prediction accuracy. The FA1 model provided a better fit than the original genotype × environment interaction model, as indicated by a higher log-likelihood (269.39 vs. 263.90) and lower AIC (-530.77 vs. -519.80) and BIC (-514.08 vs. -503.11) values. Despite the improved model fit, genotype rankings obtained from the two models were highly consistent (r = 0.968). The top-performing genotypes identified under the original model (G22, G23, G29, and G27) remained unchanged under the FA1 model, and the overall set of superior genotypes was largely maintained. These results indicate that accounting for genetic covariance among environments improved model fit, while the identification of superior and stable genotypes remained robust across covariance structures (**Table 3**).

**Table 3:** Comparison of model fit statistics and genotype ranking consistency between the original genotype × environment interaction (G × E) model and the first-order factor analytic (FA1) model for grain yield.

Parameter	Original G × E model	FA1 model
Log-likelihood	263.90	269.39
Akaike information criterion (AIC)	-519.80	-530.77
Bayesian information criterion (BIC)	-503.11	-514.08
Rank correlation (*r*)	–	0.968

Lower AIC and BIC values indicate superior model fit. Rank correlation (*r*) was calculated between predicted genotypic effects obtained from the original G × E model and the FA1 model.

### 3.4 Trait associations and distribution patterns across environments

**Figure 2:**
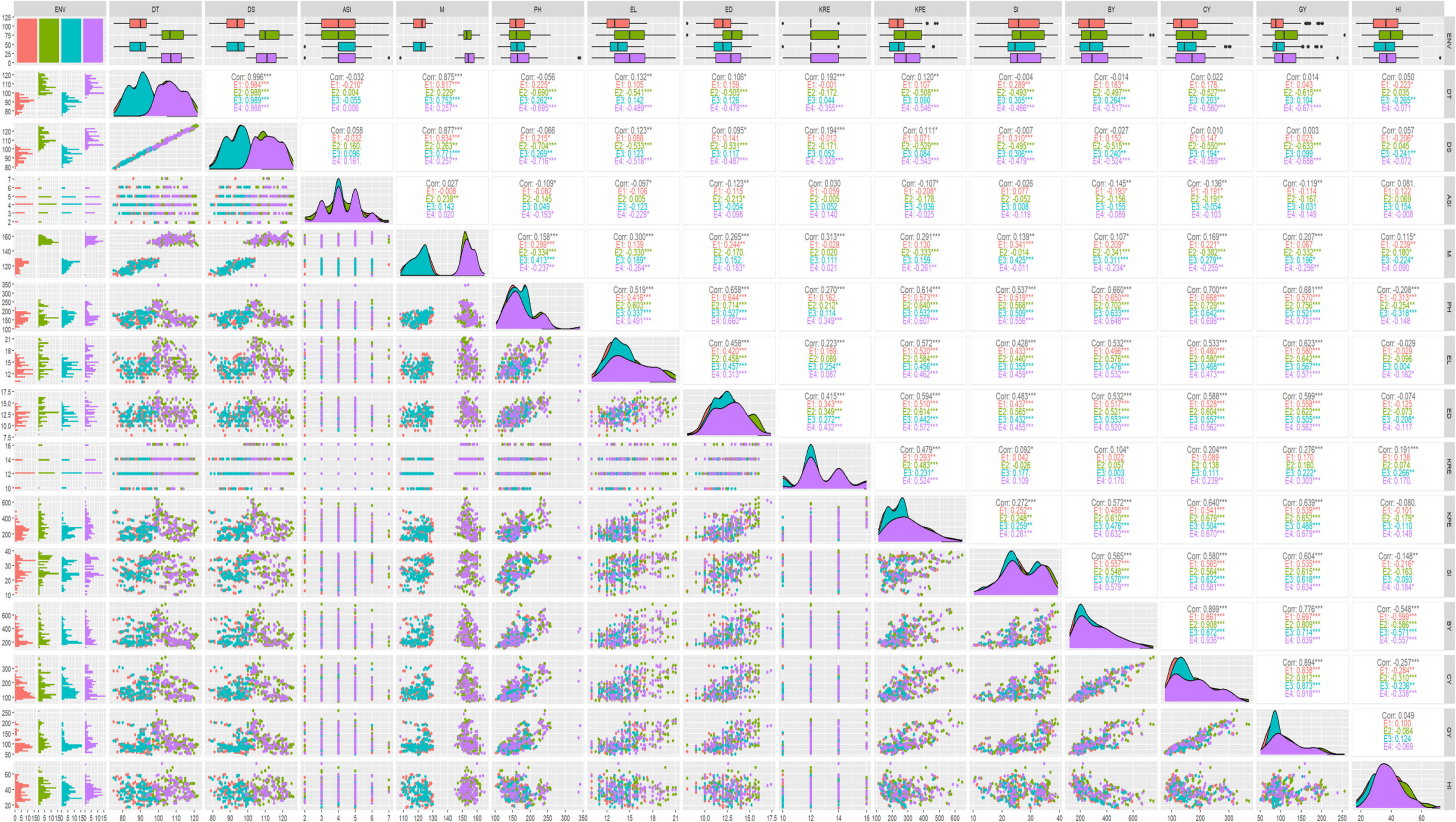
Correlations and frequency distributions of quantitative traits among 60 maize genotypes across four different environments.

The pairwise correlation matrix revealed distinct patterns of association among morphological and yield-related traits in the four environments studied. The diagonal density plots indicated that most traits followed near-normal distributions with environment-specific shifts in their central tendency, highlighting the differential environmental influence on the trait’s performance (**Figure 2**).

The grain yield exhibited strong positive associations with cob yield (0.894), biological yield (0.776), plant height (0.681), kernels per ear (0.639), ear length (0.623), seed index (0.604), and ear diameter (0.599) while showing a weak negative correlation with anthesis–silking interval (ASI, -0.119). Days to 50% tasseling correlated almost perfectly with days to 50% silking (0.996), followed by days to maturity (0.875), and non-significant negative associations with plant height (-0.056), ASI (-0.032), biological yield (-0.014), and seed index (-0.004) were shown. Days to 50% silking was strongly linked to maturity (0.877) but weakly and negatively related to plant height (-0.066) and biological yield (-0.027). Plant height correlated positively with cob yield (0.700), grain yield (0.681), biological yield (0.660), ear diameter (0.658), kernels per ear (0.614), and seed index (0.537) but negatively with harvest index (-0.208). Ear length showed positive correlations with grain yield (0.623), kernels per ear (0.572), cob yield (0.533), biological yield (0.532), and ear diameter (0.458). Ear diameter correlated positively with grain yield (0.599), kernels per ear (0.594), cob yield (0.588), biological yield (0.532), and seed index (0.483). Kernels per ear were strongly associated with cob yield (0.640), grain yield (0.639), and biological yield (0.572). Seed index correlated positively with grain yield (0.604), cob yield (0.580), and biological yield (0.565). Biological yield was strongly linked to cob yield (0.899) and grain yield (0.776) but negatively to harvest index (-0.548). Finally, cob yield correlated positively with grain yield (0.894) and negatively with harvest index (-0.257).

### 3.5 BLUP-based stability analysis and ranking of maize genotypes using WAASB and WAASBY indices

**Figure 3:**
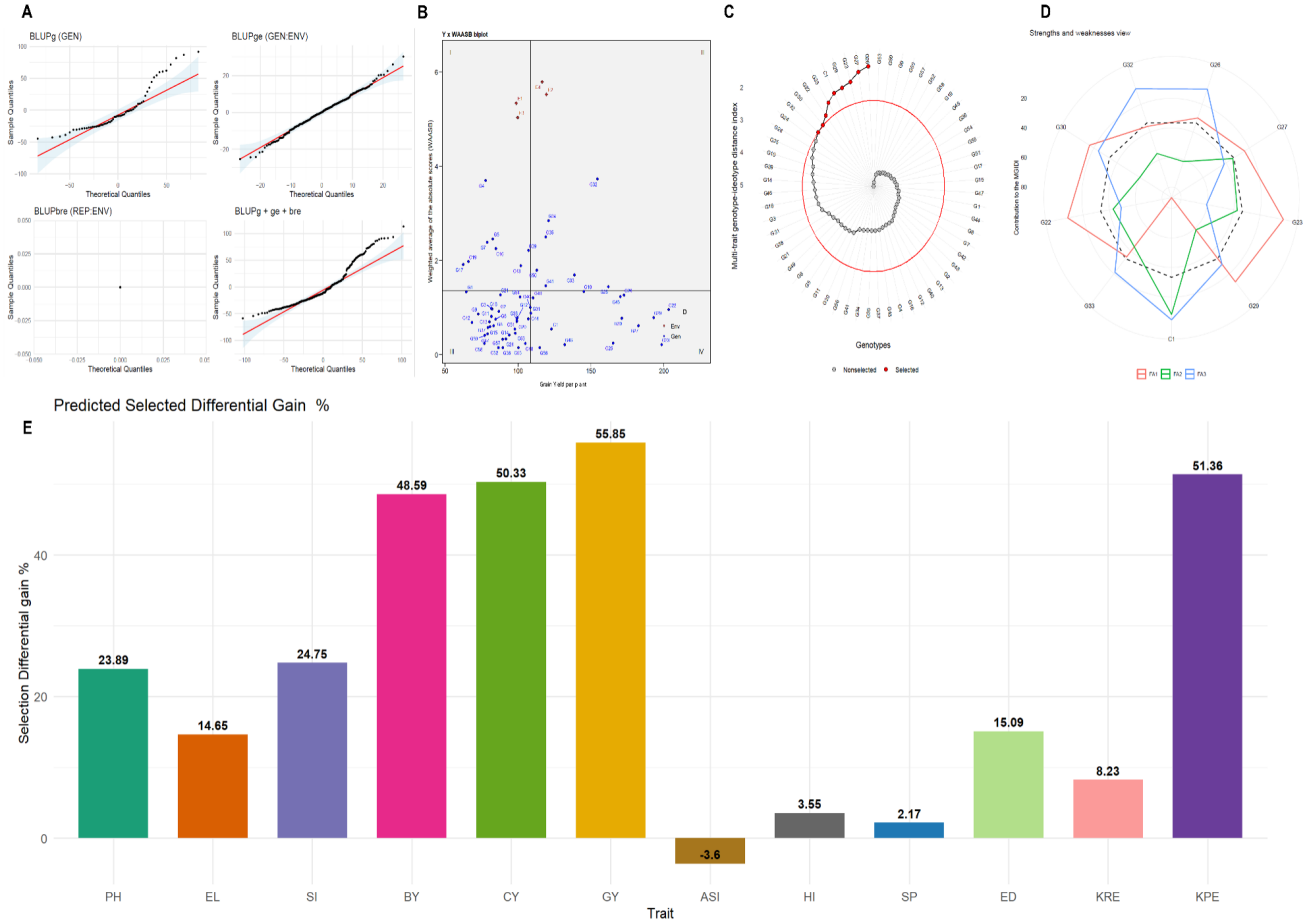
**(a)** Quantile–quantile plots illustrating the distribution of block within replication (BLUPbre), genotype (BLUPg), total phenotypic effects (BLUPg+ge+bre), and genotype-by-environment interaction (BLUPge), all depicted with a 95% confidence interval for normality adjustment across various environments. **(b)** WAASB × seed yield per plant (Y) biplot for the 60 maize genotypes across four environments. **(c)** Genotype ranking in ascending order for the MGIDI index tested under multi-environmental trials with selection intensity of 15% (red circle). The red circle represents the threshold MGIDI score, the inner circle next to the threshold circle represents the smallest value of the scale, and the innermost circle represents the highest value of the scale. **(d)** Strength and weakness view of the stable genotypes identified across four environments, shown as the proportion of each factor on the computed MGIDI index. **(e)** Percentage of predicted selection differential gain achieved in multi-environment trials through MGIDI,.

In the present study, the Q–Q plots showed that most data points closely followed the theoretical quantile line, with only minor deviations at the tails (**Figure 3A**). The distribution of different BLUP-derived effects, including genotype effects (BLUPg), genotype × environment interaction effects (BLUPge), combined genotype and genotype × environment interaction effects (BLUPg+ge), and block-within-replication effects (BLUPbre), exhibited either normal or near-normal distributions. This indicates that the assumption of normality for random effects was largely satisfied. The WAASB × Y biplot, which simultaneously depicts mean grain yield (GY, BLUP estimates) on the x-axis and the WAASB on the y-axis, was used to rank the 60 maize genotypes for productivity and stability across the four environments (**Figure 3B**). In this biplot, genotypes with high yield and low WAASB values (quadrant IV) are considered the most desirable, as they combine superior mean performance with high phenotypic stability across environments. From the biplot, genotypes G10, G22, G23, G25, G26, G27, G29, G30, G45, G46, G56, and C1 were located in quadrant IV, indicating that these genotypes exhibited above-average grain yield along with low WAASB values, reflecting stable performance across environments. Among these, G26 and G45 were positioned closest to the ideal region (high yield and lowest WAASB), confirming them as the most promising genotypes for broad adaptability and consistent productivity.

Genotypes located in quadrant II (high yield, high WAASB) exhibited above-average grain yield but showed high genotype × environment interaction, indicating instability across environments. Genotypes such as G28, G32, G33, G34, G35, and G50 were positioned in this region. These genotypes may be suitable for specific environments but are not ideal for wide adaptation. Genotypes in quadrant III (low yield, low WAASB) were relatively stable but recorded below-average grain yield. These include G1, G2, G3, G9, G11, G54, G40, and others and are therefore considered stable but less desirable for yield improvement. Genotypes located in quadrant I (low yield, high WAASB) showed both poor performance and high instability, making them the least desirable for selection. Genotypes such as G4, G5, G7, G16, G17, G19, and G43 fall into this category and should be excluded from further breeding consideration.

The WAASBY ranking, which integrates mean performance and stability, identified G10, G26, G45, G49, and G27 as the top-performing genotypes. The check (C1) was positioned in the high WAASB region or near the mean yield line, indicating comparatively lower stability than the best-performing experimental genotypes, particularly G26, G30, and G45.

### 3.6 Genotype selection across the test environments using different selection indices

A total of 60 maize genotypes including check were evaluated in four different environments. These genotypes were further evaluated using the MGIDI, MTSI, MTMPS, and FAI-BLUP indices to identify stable and high-performing maize genotypes and compare the efficiency of the different multi-trait selection approaches across environments.

#### 3.6.1 Multi-trait genotype-ideotype distance index

##### 3.6.1.1 Factor description for MGIDI

Applying the Kaiser criterion, which retains factors with eigenvalues exceeding 1, revealed three factors that together accounted for 76.06% of the total variance in the dataset, as assessed by the WAASBY value of BLUP estimates. The eigenvalues, explained variance, and factorial loadings from the MGIDI factor analysis are summarized in **Table 4**. The first factor (FA1) had an eigenvalue of 5.97 and accounted for 49.82% of the variance. The second factor (FA2) had an eigenvalue of 2.01, showing 16.75% of the variance, while the third factor (FA3) showed an eigenvalue 1.14 with 9.49% of the total variance. FA1 was primarily associated with GY (0.89), SI (0.83), CY (0.8), EL (0.78), BY (0.77), PH (-0.73), and ED (0.61). FA2 was strongly associated with SP (-0.93) and HI (-0.89), and finally, FA3 was linked with KRE (0.88), KPE (0.72), and ED (0.62). After varimax rotation, the average communality (*h*) was 76.17, with communalities for the traits ranging from 0.28 (ASI) to 0.92 (BY), indicating the proportion of each trait’s variance accounted for by the extracted factors. Uniqueness values ranged from 0.1 to 0.72, representing the variance in each trait that was not explained by the factors (**Table 4**).

**Table 4:** Factor analysis results from MGIDI evaluation of grain yield and its attributes across the four environments.

VAR	FA1	FA2	FA3	Communality	Uniqueness
ASI	0.13	0.43	0.27	0.28	0.72
PH	-0.73	-0.25	-0.4	0.75	0.25
EL	0.78	-0.04	0.17	0.64	0.36
ED	0.61	0.17	0.62	0.79	0.21
KRE	0.04	-0.18	0.88	0.81	0.19
KPE	0.46	0.24	0.72	0.79	0.21
SI	0.83	0.02	-0.06	0.69	0.31
BY	0.77	0.56	0.15	0.92	0.08
CY	0.8	0.43	0.26	0.9	0.1
GY	0.89	0.06	0.3	0.88	0.12
HI	-0.16	-0.89	0.13	0.83	0.17
SP	0.01	-0.93	0.01	0.86	0.14
Eigen values	5.97	2.01	1.14	–	–
Variance, %	49.82	16.75	9.49	–	–
CV, %	49.82	66.57	76.07	–	–

VAR, variable or trait; FA1, factor 1; FA2, factor 2; FA3, factor 3; DT, days to 50% tasseling; DS, days to 50% silking; ASI, anthesis silking interval; M, days to maturity; PH, plant height (cm); EL, ear length (cm); ED, ear diameter (cm); KRE, number of kernel rows per ear; KPE, number of kernels per ear; SI, seed index; BY, biological yield per plant (g); CY, cob yield per plant (g); GY, grain yield per plant (g); HI, harvest index; SP, shelling percentage; CV, cumulative variance (%).

#### 3.6.2 MGIDI-based genotype selection and strengths–weaknesses analysis

The genotype ranking based on the MGIDI index is presented in **Figure 3C**. Among the 60 maize genotypes evaluated, including the check, nine genotypes were selected using a selection intensity of 15%. The selected genotypes are presented in **Table 5**. These genotypes exhibited the lowest MGIDI values, indicating closer proximity to the ideotype and superior overall performance across multiple traits and environments.

**Table 5:** List of common maize genotypes identified across the different multi-trait selection indices.

Methods	Count	Name of the genotypes
MGIDI and FAI-BLUP	6	G26, G27, G23, G29, C1, G22
MGIDI and MTSI	5	G26, G27, C1, G30, G32
MGIDI and MTMPS	3	G26, G27, G23
FAI-BLUP and MTSI	3	G10, G26, G27, C1
FAI- BLUP and MTMPS	4	G23, G25, G27, G26
MTSI and MTMPS	2	G27, G26
MGIDI, MTMPS, MTSI, and FAI-BLUP	2	G26 and G27

The strengths and weaknesses of the selected genotypes are illustrated in **Figure 3D** through the proportional contribution of each factor (FA1–FA3) to the MGIDI score. In this graphical representation, the distance from the center reflects the contribution of a factor to the MGIDI value. Lower factor contributions indicate strengths, as the genotype is closer to the ideotype for the traits grouped within that factor, whereas higher contributions indicate weaknesses. The dashed circle represents the average contribution of each factor across the selected genotypes.

FA1 was primarily associated with yield-related traits, including PH, EL, BY, CY, GY, and SI. Therefore, genotypes exhibiting lower FA1 contributions demonstrated superior performance for these traits. Genotypes G26, G27, G32, and G33 showed FA1 as a strength, whereas G22, G23, G29, and G30 displayed relatively higher FA1 contributions, indicating greater scope for improvement in yield-related traits.

FA2 was mainly associated with ASI, HI, and SP. Genotypes G22, G23, and G27 exhibited lower FA2 contributions and therefore represented strengths for this group of traits. In contrast, the check genotype (C1) showed a comparatively higher FA2 contribution, indicating relatively weaker performance for traits represented by this factor.

FA3 was associated with ED, KRE, and KPE. Genotypes G27, G29, and G30 exhibited low FA3 contributions and therefore showed strengths for these traits. Conversely, C1, G26, G32, and G33 displayed higher FA3 contributions, indicating that these traits contributed more strongly to their deviation from the ideotype.

Overall, genotype G27 exhibited favorable contributions across all three factors, indicating a balanced combination of desirable agronomic performance and trait expression across environments, with minimal deviation from the ideotype. The adequacy of the factor structure was further assessed using variance explained, communality, and uniqueness estimates. The first three factors exhibited eigenvalues of 5.97, 2.01, and 1.14, accounting for 49.82%, 16.75%, and 9.49% of the total variation, respectively. These factors together explained 76.07% of the cumulative variation among traits, indicating that the factor model captured a substantial proportion of the overall variability present in the dataset.

The communality values ranged from 0.28 (ASI) to 0.92 (BY), indicating that most traits were adequately represented by the three extracted factors. Biological yield (0.92), cob yield (0.90), grain yield (0.88), shelling percentage (0.86), and harvest index (0.83) exhibited high communalities, suggesting that a large proportion of their variation was explained by the factor model. In contrast, anthesis–silking interval (ASI) exhibited the lowest communality (0.28) and highest uniqueness (0.72), indicating that much of its variation was not captured by the extracted factors and may be influenced by trait-specific effects.

#### 3.6.3 Predicted selection differential gain obtained through MGIDI

The predicted genetic gains under selection and selection differentials for WAASB of all the traits are presented in **Figure 3E**. The MGIDI index achieved a favorable selection differential for 11 traits among the traits studied. Positive selection differentials were observed for all traits except ASI (-3.60). The percent of selection differential ranged from -3.60 for ASI to 55.9 for GY (**Table 6**). The genetic gain under selection (Δ*G*%) ranged from -0.523 for the ASI trait to 50.6 for the GY. These results reflect the differential responses of traits to selection pressure and provide insights into targeted breeding programs.

**Table 6:** Selection differential and selection gain for weighted average of absolute scores (WAASB) for each factor and variable among 59 maize genotypes across the environments.

Trait	Factor	*X*_o_	*X*_s_	*S*	*S*%	Δ*G*	Δ*G*%	Indicator
ASI	FA2	4.19	4.04	-0.151	-3.60	-0.0219	-0.523	Increase
PH	FA1	167.0	207.0	40.0	14.6	35.7	21.3	decrease
EL	FA1	14.3	16.4	2.09	14.6	1.58	11.0	Increase
ED	FA3	12.6	14.3	1.88	15.1	1.46	11.8	Increase
KRE	FA3	12.6	13.7	1.04	8.23	0.650	5.16	Increase
KPE	FA3	271.0	410.0	139.0	51.4	119.0	43.70	Increase
SI	FA1	26.7	33.3	6.60	24.8	6.46	24.20	Increase
BY	FA1	305.0	453.0	148.0	48.6	144.0	47.30	Increase
CY	FA1	165.0	248.0	83.1	50.3	77.90	47.20	Increase
GY	FA1	109.0	169.0	60.7	55.9	55.0	50.6	Increase
HI	FA2	37.9	39.2	1.35	3.55	1.17	3.10	Increase
SP	FA2	67.2	68.7	1.46	2.17	1.24	1.84	Increase

*X*_o_, population mean; *X*_s_, mean of selected individual; *S*, selection differential; *S*%, selection differential percentage; G, selection gain; Δ*G*%, selection gain percentage; FA1, factor 1; FA2, factor 2; FA3, factor 3; DT, days to 50% tasseling; DS, days to 50% silking; ASI, anthesis silking interval; M, days to maturity; PH, plant height (cm); EL, ear length (cm); ED, ear diameter (cm); KRE, number of kernel rows per ear; KPE, number of kernels per ear; SI, seed index; BY, biological yield per plant (g); CY, cob yield per plant (g); GY, grain yield per plant (g); HI, harvest index; SP, shelling percentage.

### 3.7 Coincidence index and common genotypes selected from the different multi-trait-based stability models

**Figure 4:**
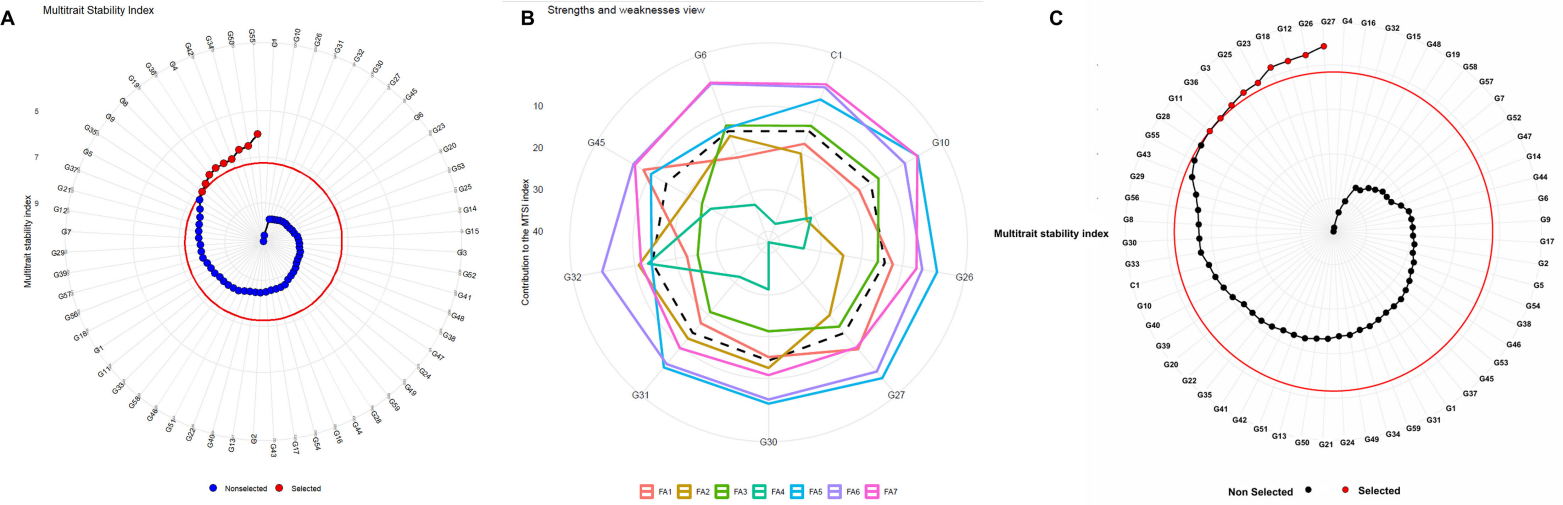
**(a)** Genotype ranking in ascending order for the MTSI index tested across environmental trials. **(b)** Strength and weakness view of the stable genotypes identified across four environments. **(c)** Selection of maize genotypes based on the MTMPS index at 15% selection intensity. The red points indicate selected genotypes, the black points indicate non-selected genotypes, and the red circle represents the selection threshold.

**Figure 5:**
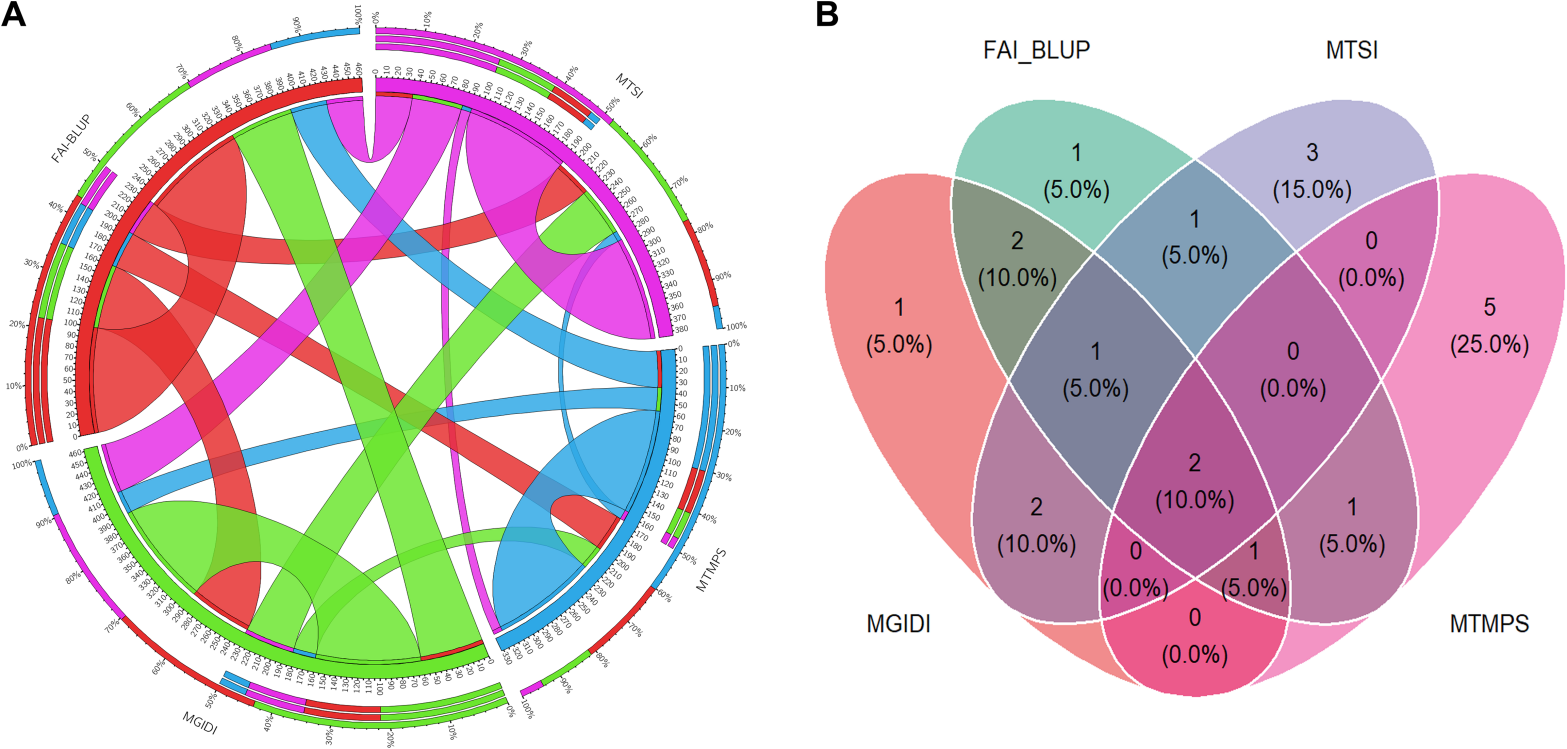
**(a)** Circos plot representing the coincidence index among the various multi-trait stability models. **(b)** Venn diagrams showing the number of common maize genotypes selected across models.

Several multi-trait stability models, such as MGIDI, MTSI (**Figures 4A, B**), MTMPS (**Figure 4C**), and FAI-BLUP, were used to select maize genotypes with 15% selection intensity. The genotypes that were selected based on the MGIDI, MTSI, MTMPS, and FAI-BLUP methods are given in **Table 6**. A comparison of these models is illustrated through a Venn diagram representing (**Figure 5A**) the common genotypes across the different model and coincidence index through the circus plot (**Figure 5B**). Among the pairwise comparisons of selection methods, MGIDI and FAI-BLUP showed the highest overlap with six common genotypes, corresponding to a coincidence index of 66.67% (**Figure 5B**). This was followed by MGIDI and MTSI with five common genotypes (55.56%) and FAI-BLUP and MTSI with four common genotypes (44.45%). MGIDI and MTMPS, as well as FAI-BLUP and MTMPS, shared three common genotypes (33.34%). The lowest overlap was observed between MTMPS and MTSI, which shared only two common genotypes with a coincidence index of 22.23%. In this study, two maize genotypes (G26 and G27) were consistently identified across all four selection models, demonstrating a strong agreement among the different multi-trait selection approaches.

## 4 Discussion

### 4.1 Mean performance and genotypic variation across environments

The pooled ANOVA revealed highly significant differences (*p* < 0.0001) among maize genotypes for all studied traits, alongside significant environment and GEI effects, indicating that both genetic and environmental factors played important roles in shaping the phenotypic expression. This finding is consistent with earlier reports in maize, where the G × E interaction has been recognized as a major challenge in identifying stable, high-yielding genotypes across diverse environments (Yan and Kang, 2003; Badu-Apraku et al., 2012). The wide range of variation observed for traits such as days to 50% tasseling (87.71–105.19 days), days to 50% silking (91.35–109.55 days), plant height (103.00–256.25 cm), grain yield (51.00–256.00 g/plant), and number of kernels per ear (112–645) reflects the broad genetic base of the 60 maize genotypes evaluated, offering ample scope for the selection and improvement of these traits. The ASI of 4.02 days and its variation from 2.00 to 7.00 days is agronomically significant, as a shorter ASI is often associated with better synchrony of male and female flowering and improved kernel set, particularly under stress conditions (Bolanos and Edmeades, 1996). The days to maturity ranged from 127.62 to 142.37 days, with a mean of 136.32 days, indicating a range from early to medium-late maturity among the genotypes, which is valuable for diversifying varietal options across cropping seasons. The wide variation in yield-related traits, such as ear length, ear diameter, seed index, biological yield, cob yield, and harvest index, underscores the diversity present in the evaluated germplasm. Low to moderate CV for most traits, including days to silking, ear length, and plant height, indicated reliable experimental conditions and data quality, whereas higher CV values for the number of KPE and ASI suggested greater environmental sensitivity for these traits, a finding substantiated by Banziger et al. (2000).

### 4.2 Variance components, heritability, and genetic parameters

The likelihood ratio tests confirmed significant genotypic effects for all traits, validating the substantial genetic variability among the tested maize genotypes. The remarkably high broad-sense heritability (*h*²_bs_) estimates were recorded for plant height (99.20%), biological yield (97.10%), cob yield (92.60%), grain yield (92.40%), and harvest index (88.00%) reflecting strong genetic control, with comparatively minor contributions from environmental influences over these traits and suggesting that selection based on phenotypic performance would be highly effective (Falconer and Mackay, 1996; Menkir et al., 2003). The slightly lower, though still substantial, heritability for KPE (79.00%) suggests that while genetic factors remain dominant, environmental conditions may exert a more pronounced influence compared to other traits. These values are in close agreement with those of previous studies in maize, which reported high heritability for yield and morphological traits (Wassom et al., 2008; Akinwale et al., 2011). Moderate to high heritability was observed for ear diameter (69.60%), ear length (68.60%), and number of kernel rows per ear (63.10%), while seed index showed moderate heritability (45.00%). Moderate heritability for these traits suggests that both genetic and non-genetic factors contribute to their expression, and multi-environment evaluation is necessary before reliable selection can be practiced (Badu-Apraku et al., 2012). In contrast, ASI recorded the lowest heritability (12.70%), indicating that this trait is predominantly governed by non-genetic factors and is highly sensitive to the prevailing environmental conditions, especially the high moisture and salt stress during the flowering period. This is consistent with the findings of Ribaut et al. (1996) and Bolanos and Edmeades (1996), who reported that ASI is a highly environment-dependent trait and should be evaluated under target stress conditions rather than across diverse environments.

The phenotypic variance (*σ*²_p_) was predominant over the genotypic variance (*σ*²_g_) for most traits, confirming the predominance of non-additive genetic effects. Consequently, phenotypic selection becomes less efficient for these traits, as environmental noise obscures the true genetic potential of genotypes. The genotype × environment interaction ratio (GEI*r*²) was highest for ear length (0.259) and harvest index (0.170), indicating that these traits are more environmentally sensitive and should be evaluated across multiple environments before selection. In contrast, traits such as biological yield (0.025), plant height (0.015), and ASI (0.010) showed low GEI contributions, suggesting a more stable expression across environments. The selection accuracy (AS) was notably high for most traits, ranging from 0.424 for seed index to 0.997 for harvest index and biological yield, reflecting the precision of the BLUP-based estimation in capturing true genetic merit (Mrode, 2014). The CV ratio exceeding unity for biological yield (15.50), KPE (8.03), cob yield (7.83), seed index (7.27), and grain yield (5.23) confirmed greater genetic variability relative to residual variation for these traits, making them favorable targets for selection. Genotypic correlations across environments (rge) were high for KPE (0.909), biological yield (0.860), and ear length (0.823), indicating a consistent ranking of genotypes for these traits regardless of the environment, while low rge values for traits such as ASI (0.012), harvest index (0.0008), plant height (0.200), and seed index (0.100) highlight the importance of multi-environment testing for these traits.

### 4.3 Trait associations and implications for indirect selection

Correlation studies among yield and yield-attributing traits are important in understanding the nature and magnitude of association among traits and provide a basis for indirect selection (Akinwale et al., 2011). In the present study, grain yield per plant showed a significant positive correlation with cob yield (0.894), biological yield (0.776), plant height (0.681), number of kernels per ear (0.639), ear length (0.623), seed index (0.604), and ear diameter (0.599). These positive associations indicate that genotypes with taller plants, longer and wider ears, greater number of kernels, and heavier seeds tend to record a higher grain yield. Similar positive associations between grain yield and these component traits have been reported in previous studies on maize (Adesoji et al., 2015; Tshikunde et al., 2019). The strong positive correlation between grain yield and cob yield (0.894) suggests that cob yield is a reliable indirect selection criterion for grain yield improvement in maize.

Days to 50% tasseling showed a strong positive correlation with days to 50% silking (0.996) and days to maturity (0.875), indicating that late-flowering genotypes tended to have a longer crop duration. The non-significant negative correlations of days to tasselling with plant height (-0.056), ASI (-0.032), and biological yield (-0.014) suggest that flowering duration has little direct influence on vegetative growth and biomass accumulation in the present germplasm line. A significant negative correlation was observed between biological yield and harvest index (-0.548) and between plant height and harvest index (-0.208), indicating that genotypes accumulating higher vegetative biomass tend to show lower efficiency in partitioning dry matter into grain. This inverse relationship between vegetative growth and harvest index is a well-known limitation in cereal breeding and underscores the need for simultaneous selection for both biomass and partitioning efficiency when developing high-yielding genotypes (Donald, 1968; Tshikunde et al., 2019). The negative correlation between ASI and grain yield (-0.119), although modest, is consistent with the established understanding that genotypes with shorter ASI tend to have better kernel set and higher grain yield, particularly under stress conditions (Bolanos and Edmeades, 1996; Ribaut et al., 1996).

### 4.4 BLUP-based stability analysis using WAASB and WAASBY indices

The WAASB × Y biplot provides a visually intuitive and statistically robust framework for the simultaneous evaluation of mean performance and phenotypic stability in multi-environment trials, overcoming the limitation of classical stability parameters that assess yield and stability independently (Olivoto et al., 2019). The classification of genotypes into four regions based on the intersection of mean grain yield and WAASB reference lines reflects the dual breeding objective of identifying genotypes that combine high productivity with broad adaptation, which is essential for cultivar development (Yan and Kang, 2003).

In the present study, genotypes G26 and G27 were positioned in quadrant IV (high yield, low WAASB), close to the ideal ideotype of maximum yield and minimum instability, indicating superior and stable performance across environments. Their consistent selection across all four multi-trait selection indices (MGIDI, MTSI, MTMPS, and FAI-BLUP) further validates their robustness and adaptability under contrasting seasonal conditions.

The relatively large number of genotypes located in quadrant II (high yield, high WAASB) indicates that several high-yielding genotypes exhibited a strong genotype × environment interaction, resulting in inconsistent performance across environments. This suggests that their yield potential is environment-specific, likely influenced by variations in temperature and photoperiod between summer and winter seasons. From a breeding perspective, reliance solely on mean yield could lead to the selection of such unstable genotypes. The WAASB component of the WAASBY index effectively penalizes these genotypes by incorporating their GEI contribution, thereby enabling more reliable selection decisions (Olivoto et al., 2019; Yue et al., 2022b).

Genotypes located in quadrant III (low yield, low WAASB) exhibited relatively stable performance but had below-average yield. Although these genotypes are not suitable for direct selection as high-yielding cultivars, they may serve as valuable genetic resources in breeding programs aimed at improving stability when crossed with high-yielding genotypes (Falconer and Mackay, 1996; Badu-Apraku et al., 2012).

In contrast, genotypes in quadrant I (low yield, high WAASB) combined poor performance with high instability, making them undesirable for further advancement. Their limited yield potential and inconsistent performance across environments indicate low breeding value under the tested conditions. The equal weighting of mean performance and stability in the WAASBY index applied in this study aligns with recommendations for selecting genotypes with broad adaptability (Olivoto et al., 2019). However, weighting can be adjusted depending on breeding objectives; greater emphasis on yield may be suitable for favorable environments, whereas prioritizing stability is advantageous under variable or stress-prone conditions (Yue et al., 2022b). The superior ranking of G26 and G27 compared to the check (C1) further highlights their potential as elite genotypes, warranting advancement to multi-location trials and possible commercial utilization (Choudhary et al., 2019; Nagesh et al., 2025).

### 4.5 Multi-trait selection indices: MGIDI, MTSI, MTMPS, and FAI-BLUP

Multi-trait selection indices represent a powerful advancement over single-trait selection approaches, enabling breeders to simultaneously improve multiple agronomically important traits while accounting for trait interrelationships and environmental stability. In this study, four complementary multi-trait selection models, namely, MGIDI, MTSI, MTMPS, and FAI-BLUP, were employed to identify elite maize genotypes with superior performance and broad adaptability.

#### 4.5.1 MGIDI factor structure and selection gains

The MGIDI analysis, based on the Kaiser criterion, retained three factors accounting for 76.06% of the total variance, demonstrating efficient dimensional reduction of the trait space. The first factor (FA1), with an eigenvalue of 5.97 explaining 49.82% of the variance, had high positive loadings for grain yield (0.89), seed index (0.83), cob yield (0.80), ear length (0.78), biological yield (0.77), and ear diameter (0.61) and a negative loading for plant height (-0.73). This grouping of yield and yield component traits in FA1 is biologically consistent, as these traits collectively determine the final grain productivity of a genotype (Olivoto and Nardino, 2021). The negative loading of plant height in FA1 suggests that within the present germplasm, taller genotypes did not necessarily record higher grain yield, which may be attributed to the trade-off between vegetative growth and dry matter partitioning into grains, as reflected in the significant negative correlation between plant height and harvest index (-0.208) observed in the present study. It suggests that moderate plant height could be desirable, as excessive vegetative growth may divert assimilates away from reproductive structures.

The second factor (FA2), with an eigenvalue of 2.01 accounting for 16.75% of the variance, had high negative loadings for shelling percentage (-0.93) and harvest index (-0.89). As higher values of these traits are desirable, their negative loadings in FA2 indicate that genotypes closer to the ideotype in this factor group have higher shelling percentage and harvest index, reflecting superior dry matter partitioning efficiency. The clustering of shelling percentage and harvest index within FA2 highlights their close interrelationship, both being indicators of efficiency in converting biological yield into economic yield. The third factor (FA3), with an eigenvalue of 1.14 explaining 9.49% of the variance, was associated with the number of kernel rows per ear (0.88), number of kernels per ear (0.72), and ear diameter (0.62). The strong positive loadings suggest that these traits are closely interrelated, with improvements in kernel row number and ear diameter contributing to higher kernel counts per ear. These traits collectively define the ear-filling capacity of a genotype and determine the potential for kernel number per ear, which is an important yield component in maize breeding (Adesoji et al., 2015). The average communality of 76.17% following varimax rotation, with trait-level communalities ranging from 0.28 (ASI) to 0.92 (biological yield), confirms that the extracted factors adequately captured the genetic variation for most traits, except for ASI, which showed high uniqueness (0.72) owing to its strong environmental sensitivity.

#### 4.5.2 Strength and weakness profiling of selected genotypes

The strengths and weaknesses view derived from the MGIDI provided genotype-specific trait group diagnostic profiles, offering mechanistic insights into the genetic architecture of each selected genotype that is directly useful for designing crossing programs (Olivoto and Nardino, 2021). Genotypes G26, G27, G32, and G33 showed FA1 as a strength, indicating superior performance for yield and yield-contributing traits, including grain yield, seed index, cob yield, ear length, and biological yield. Genotypes G22, G23, G29, and G30 showed weakness for FA1-associated traits, suggesting that they are relatively inferior for these yield-related characters. For FA2, associated with shelling percentage and harvest index, genotypes G22, G23, and G27 showed strength, indicating better dry matter partitioning efficiency, while genotype C1 showed weakness for FA2-associated traits. For FA3, associated with ear morphological traits including ear diameter, number of kernel rows per ear, and number of kernels per ear, genotypes G27, G29, and G30 showed strength, while C1, G26, G32, and G33 showed weakness, indicating lower ear-filling capacity for these genotypes.

This strength and weakness profiling has direct implications for the hybridization strategy. Genotype G26, which shows strength in FA1 (yield traits) but weakness in FA3 (ear morphology traits), could be crossed with genotypes strong in FA3, such as G27, G29, or G30, to combine superior yield attributes with better ear morphology in the segregating generations (Donald, 1968; Badu-Apraku et al., 2012). The consistent weakness of several selected genotypes for FA2-associated traits (harvest index and shelling percentage) indicates that dry matter partitioning efficiency is a limiting factor in the current germplasm pool, and deliberate selection for these traits in future breeding cycles is warranted (Menkir et al., 2003).

### 4.6 Selection gain and selection differential under MGIDI

Based on a 15% selection intensity, nine genotypes were selected from the 60 evaluated genotypes using the MGIDI. The MGIDI achieved favorable selection differentials for 11 out of 12 traits, with positive selection differentials observed for all traits except ASI (-3.60%), for which a negative differential is desirable since a lower ASI is the breeding objective. The selection differential for grain yield was the highest (55.9%), indicating that the selected genotypes were substantially superior in grain yield compared to the population mean, confirming the effectiveness of the MGIDI in directing selection pressure toward high-yielding genotypes (Olivoto and Nardino, 2021). The predicted genetic gain under selection (Δ*G*%) ranged from -0.523% for ASI to 50.6% for grain yield. These estimates reflect the expected genetic improvement in the next generation if the selected genotypes are used as parents, and the relatively high predicted gains for grain yield and yield component traits demonstrate the breeding potential of the selected germplasm (Bates et al., 2015; Falconer and Mackay, 1996).

The negative selection differential for ASI (-3.60%) and the corresponding negative genetic gain (-0.523%) indicate that MGIDI successfully identified genotypes with shorter ASI relative to the population mean, which is a desirable outcome in maize breeding, as shorter ASI is associated with better synchrony between pollen shed and silk receptivity and improved grain set under stress conditions (Bolanos and Edmeades, 1996). However, given the low heritability of ASI (12.70%) and its very low genotypic correlation across environments (rge = 0.012) observed in the present study, breeding progress for ASI through phenotypic selection alone is expected to be limited, and the use of molecular markers associated with ASI would complement phenotypic selection for this trait (Ribaut et al., 1996; Banziger et al., 2000).

### 4.7 Comparison of multi-trait selection indices and coincidence of selected genotypes

The use of four multi-trait selection indices, MGIDI, MTSI, MTMPS, and FAI-BLUP, with a uniform 15% selection intensity allowed a direct comparison of their selection efficiency and the degree of agreement among them. The highest coincidence was observed between MGIDI and FAI-BLUP (66.67%, six common genotypes), followed by MGIDI and MTSI (55.56%, five common genotypes) and FAI-BLUP and MTSI (44.45%, five genotypes). These high coincidence values reflect methodological similarity between MGIDI and FAI-BLUP in relying on BLUP estimates and genotype–ideotype distance for selection, consistent with the observations of Rocha et al. (2017).

The moderate coincidence between MGIDI and MTSI (55.56%) can be attributed to the methodological difference between the two indices: MGIDI selects genotypes based on their distance from the ideotype using BLUP mean values alone, while MTSI incorporates both mean performance and phenotypic stability (WAASB) into the factor matrix, thereby penalizing genotypes with high mean performance but inconsistent behavior across environments (Olivoto and Nardino, 2021). Consequently, genotypes selected by MGIDI but not by MTSI are likely to be high-yielding but less stable, whereas genotypes selected by MTSI but not by MGIDI may be more stable but slightly inferior in terms of mean performance.

The lower coincidence of MTMPS with all other indices (22.23% to 33.34%) is explained by its use of Wricke’s ecovalence (Wi) as the stability measure rather than the WAASB index. Wricke’s ecovalence quantifies the contribution of each genotype to the total GEI sum of squares, and genotypes with lower Wi values are considered more stable (Bates et al., 2015). Since ecovalence and WAASB capture different dimensions of phenotypic stability, the selection sets derived from MTMPS and the other three indices are expected to differ, as observed in the present study. Despite these methodological differences, the minimum coincidence between MTMPS and MTSI (22.23%, two common genotypes) suggests that these two indices differ most in their selection criteria and that genotypes selected by both indices simultaneously would represent a particularly conservative and stable selection (Yan and Kang, 2003).

Importantly, genotypes G26 and G27 were consistently identified across all four multi-trait selection indices, demonstrating a strong cross-model agreement on the superiority of these two genotypes. Their consistent selection, regardless of the stability measure or weighting scheme used, confirms that G26 and G27 possess high mean performance combined with broad phenotypic stability for the traits evaluated in the present study. The identification of common genotypes across different multi-trait selection methods is considered strong evidence of their genetic superiority and is the most reliable criterion for advancing genotypes to higher stages of evaluation (Yue et al., 2022b; Olivoto and Nardino, 2021; Rocha et al., 2017). These genotypes are therefore recommended as elite candidates for multi-location evaluation and for use as parents in future hybridization programs aimed at improving grain yield and stability in maize.

## 5 Conclusions

The integration of multi-environment testing, variance component analysis, BLUP-based stability estimation, and multi-trait selection indices in this study offers a comprehensive and statistically rigorous framework for maize improvement under variable agro-climatic conditions. The evaluation across four environments spanning two summer and two winter seasons at Acharya Narendra Deva University of Agriculture and Technology, Ayodhya, provided meaningful temporal replication, enabling the capture of season-specific and year-specific GEI effects through the fitted random-effects model. The identification of genotypes with high broad-sense heritability for key yield traits confirms that phenotypic selection in structured multi-environment trials can effectively capture genetic merit for these traits. The MGIDI-based selection differential and higher genetic gain estimates, for grain yield, highlight the significant breeding value present in the evaluated germplasm and the potential for rapid genetic improvement through targeted selection. The elite genotypes identified in the present study, particularly G26 and G27, should be validated in multi-location trials spanning diverse agro-climatic zones before deployment recommendations are made for broader regions. Future research should focus on the molecular characterization of these elite genotypes to elucidate the genomic basis of their superior performance and stability, enabling marker-assisted introgression of their favorable alleles into locally adapted backgrounds. Additionally, expanding the evaluation to a broader network of environments, including stress-prone locations, would further validate the broad adaptability of these genotypes and inform targeted deployment strategies aligned with specific agro-ecological zones.

## Data Availability

The original contributions presented in the study are included in the article/**Supplementary Material**. Further inquiries can be directed to the corresponding authors.
