# Supplementary material for: Identification of stable and high-yielding maize genotypes using BLUP-based stability and multi-trait selection indices across diverse environments
Source: Front Plant Sci. 2026 Jul 21;17:1858657. doi: 10.3389/fpls.2026.1858657 (PMC13433520; doi:10.3389/fpls.2026.1858657)
Supplement: Supplementary file 1 [file Table1.docx]

**Table S1. List of 60 Maize genotypes used on the present study**

| **S.N.** | **Genotypes** | **Pedigree** | **Source** | **Code** | **S.N.** | **Genotypes** | **Pedigree** | **Source** | **Code** |
| --- | --- | --- | --- | --- | --- | --- | --- | --- | --- |
| **1** | VL118563 | - | BHU | G1 | **31** | UMC-13 | composite | Behraich | G31 |
| **2** | VL156016 | - | BHU | G2 | **32** | UMC-16 | composite | Behraich | G32 |
| **3** | HUZM242 | BH-3309 | BHU | G3 | **33** | UMC-17 | composite | Behraich | G33 |
| **4** | HUZM246 | BH-3447 | BHU | G4 | **34** | UMC-30 | composite | Behraich | G34 |
| **5** | HUZM-V994U-24 | - | BHU | G5 | **35** | SH-9701 |  | Behraich | G35 |
| **6** | HKI-327T | - | BHU | G6 | **36** | HUZM-536 | - | BHU | G36 |
| **7** | HKI-1015-WG-8 | - | BHU | G7 | **37** | CML-451 | - | BHU | G37 |
| **8** | CML-141 | - | BHU | G8 | **38** | HUZM-185 | - | BHU | G38 |
| **9** | JCY3-7-1-2-1-b-1-2-2-1 | - | BHU | G9 | **39** | VQL-1 | - | BHU | G39 |
| **10** | DKC-9081 | - | BHU | G10 | **40** | CML-563 | - | BHU | G40 |
| **11** | HUZM227 | - | BHU | G11 | **41** | CML-14137 | - | BHU | G41 |
| **12** | NDM-1 | - | BHU | G12 | **42** | VL-109179 | - | BHU | G42 |
| **13** | NDM-2 | - | BHU | G13 | **43** | NDM-8 | Collection | Bihar | G43 |
| **14** | NDM-3 | - | BHU | G14 | **44** | NDM-9 | Collection | Bihar | G44 |
| **15** | NDM-4 | - | BHU | G15 | **45** | NDM-10 | Collection | Bihar | G45 |
| **16** | NDM-5 | - | BHU | G16 | **46** | NDM-11 | Collection | Bihar | G46 |
| **17** | NDM-6 | - | BHU | G17 | **47** | NDM-12 | Collection | Bihar | G47 |
| **18** | NDM-7 | - | BHU | G18 | **48** | NDM-13 | Collection | Bihar | G48 |
| **19** | LM-13 | - | BHU | G19 | **49** | NDM-14 | Collection | Bihar | G49 |
| **20** | DMRQPM-58-1 | - | BHU | G20 | **50** | NDM-15 | Collection | Bihar | G50 |
| **21** | DMRQPM-58-2 | - | BHU | G21 | **51** | NDM-16 | Collection | Bihar | G51 |
| **22** | UMC-1 | composite | Behraich | G22 | **52** | NDM-17 | Collection | Bihar | G52 |
| **23** | UMC-8 | composite | Behraich | G23 | **53** | NDM-18 | Collection | Bihar | G53 |
| **24** | UMC-9 | composite | Behraich | G24 | **54** | NDM-19 | Collection | Bihar | G54 |
| **25** | UMC-10 | composite | Behraich | G25 | **55** | NDM-20 | Collection | Bihar | G55 |
| **26** | UMC-12 | composite | Behraich | G26 | **56** | NDM-21 | Collection | Bihar | G56 |
| **27** | UMC-14 | composite | Behraich | G27 | **57** | NDM-22 | Collection | Bihar | G57 |
| **28** | UMC-15 | composite | Behraich | G28 | **58** | NDM-23 | Collection | Bihar | G58 |
| **29** | UMC-18 | composite | Behraich | G29 | **59** | NDM-24 | Collection | Bihar | G59 |
| **30** | UMC-20 | composite | Behraich | G30 | 60 | **Check** |  |  |  |

**Table S2. Pooled average representation of 60 maize genotypes for 15 traits across locations**

| **Genotypes** | **DT** | **DS** | **ASI** | **M** | **PH** | **EL** | **ED** | **KRE** | **KPE** | **SI** | **BY** | **CY** | **GY** | **HI** | **SP** |
| --- | --- | --- | --- | --- | --- | --- | --- | --- | --- | --- | --- | --- | --- | --- | --- |
| **C1** | 87.71 | 91.35 | 3.51 | 130.68 | 176.24 | 16.39 | 14.34 | 13.73 | 466.89 | 27.27 | 222.44 | 160.23 | 122.82 | 55.21 | 76.64 |
| **G1** | 102.22 | 106.79 | 4.27 | 139.05 | 113.02 | 12.28 | 11.12 | 12.71 | 250.61 | 19.74 | 149.80 | 95.10 | 64.28 | 42.91 | 67.59 |
| **G2** | 94.34 | 98.23 | 3.83 | 133.29 | 115.39 | 11.46 | 11.22 | 11.21 | 160.14 | 23.88 | 152.34 | 110.76 | 86.77 | 56.96 | 78.34 |
| **G3** | 99.54 | 103.59 | 3.94 | 137.92 | 163.41 | 14.51 | 12.28 | 11.96 | 300.19 | 24.30 | 158.73 | 100.01 | 81.70 | 51.47 | 81.69 |
| **G4** | 94.22 | 98.35 | 3.94 | 132.27 | 150.16 | 14.37 | 11.71 | 12.23 | 153.30 | 18.79 | 193.54 | 101.22 | 75.50 | 39.01 | 74.59 |
| **G5** | 93.94 | 98.60 | 4.52 | 131.50 | 174.50 | 14.26 | 12.21 | 12.00 | 254.88 | 26.84 | 214.43 | 114.81 | 82.06 | 38.27 | 71.48 |
| **G6** | 96.63 | 100.49 | 3.80 | 134.37 | 133.32 | 14.93 | 11.99 | 12.71 | 137.73 | 28.47 | 198.92 | 107.72 | 84.39 | 42.42 | 78.34 |
| **G7** | 98.26 | 102.63 | 4.29 | 137.04 | 117.31 | 13.51 | 10.00 | 12.00 | 220.31 | 19.45 | 184.50 | 101.59 | 78.29 | 42.44 | 77.07 |
| **G8** | 104.28 | 108.75 | 4.41 | 138.24 | 156.17 | 16.82 | 10.12 | 11.21 | 173.92 | 24.49 | 190.61 | 105.60 | 83.01 | 43.55 | 78.61 |
| **G9** | 100.93 | 105.19 | 4.17 | 141.04 | 147.54 | 12.48 | 11.43 | 10.47 | 143.32 | 32.84 | 200.48 | 104.07 | 72.54 | 36.19 | 69.71 |
| **G10** | 96.22 | 100.43 | 3.96 | 137.58 | 163.09 | 13.81 | 12.31 | 12.47 | 171.33 | 29.89 | 239.11 | 189.31 | 143.77 | 60.12 | 75.94 |
| **G11** | 99.93 | 104.80 | 4.84 | 139.09 | 156.45 | 13.42 | 11.90 | 12.67 | 149.66 | 29.37 | 175.64 | 107.47 | 81.78 | 46.56 | 76.09 |
| **G12** | 101.98 | 107.03 | 4.86 | 141.42 | 145.70 | 15.13 | 12.39 | 12.00 | 120.81 | 34.31 | 217.33 | 124.41 | 99.38 | 45.73 | 79.88 |
| **G13** | 99.77 | 103.74 | 3.94 | 138.57 | 142.02 | 13.46 | 12.14 | 14.68 | 331.36 | 11.18 | 168.32 | 104.39 | 80.33 | 47.73 | 76.95 |
| **G14** | 92.75 | 98.04 | 5.17 | 132.59 | 134.76 | 14.18 | 10.73 | 14.44 | 345.42 | 26.64 | 233.91 | 122.54 | 93.83 | 40.12 | 76.57 |
| **G15** | 98.11 | 102.18 | 3.96 | 130.84 | 124.07 | 12.52 | 9.64 | 12.00 | 150.23 | 12.97 | 171.67 | 97.90 | 79.51 | 46.32 | 81.22 |
| **G16** | 96.37 | 101.06 | 4.52 | 132.72 | 140.16 | 12.79 | 10.79 | 12.71 | 150.61 | 29.02 | 186.44 | 123.99 | 84.46 | 45.30 | 68.12 |
| **G17** | 100.47 | 105.10 | 4.38 | 139.38 | 129.78 | 11.91 | 11.22 | 13.21 | 202.75 | 15.84 | 142.88 | 95.28 | 62.28 | 43.59 | 65.36 |
| **G18** | 103.69 | 108.58 | 4.81 | 136.66 | 156.70 | 13.41 | 13.51 | 14.20 | 283.93 | 22.08 | 177.64 | 106.45 | 82.13 | 46.23 | 77.15 |
| **G19** | 99.69 | 103.67 | 3.77 | 140.78 | 159.96 | 12.14 | 10.55 | 12.00 | 191.68 | 24.59 | 205.46 | 101.47 | 65.82 | 32.04 | 64.87 |
| **G20** | 97.77 | 101.53 | 3.69 | 135.16 | 129.00 | 12.22 | 12.36 | 13.47 | 216.48 | 20.86 | 211.88 | 138.74 | 99.04 | 46.74 | 71.38 |
| **G21** | 98.05 | 103.01 | 4.82 | 140.32 | 159.75 | 10.97 | 12.19 | 13.73 | 213.95 | 21.30 | 171.96 | 118.35 | 87.90 | 51.12 | 74.27 |
| **G22** | 88.35 | 92.52 | 4.05 | 131.32 | 203.99 | 17.62 | 14.07 | 12.71 | 431.92 | 35.33 | 570.74 | 300.56 | 203.17 | 35.60 | 67.60 |
| **G23** | 98.95 | 102.96 | 3.88 | 139.05 | 211.99 | 17.45 | 14.18 | 12.96 | 372.37 | 33.50 | 468.76 | 279.52 | 198.33 | 42.31 | 70.95 |
| **G24** | 94.99 | 99.74 | 4.64 | 139.80 | 208.46 | 13.43 | 13.68 | 14.68 | 309.90 | 34.39 | 272.39 | 134.81 | 91.37 | 33.54 | 67.78 |
| **G25** | 94.18 | 98.81 | 4.54 | 137.56 | 216.37 | 17.38 | 12.70 | 12.47 | 277.17 | 35.10 | 376.10 | 210.07 | 164.85 | 43.83 | 78.47 |
| **G26** | 96.47 | 100.11 | 3.41 | 136.93 | 214.94 | 18.25 | 14.80 | 14.68 | 397.37 | 33.40 | 442.53 | 249.87 | 171.61 | 38.78 | 68.68 |
| **G27** | 95.17 | 99.05 | 3.80 | 136.66 | 207.61 | 15.25 | 14.14 | 14.00 | 308.88 | 34.80 | 417.02 | 254.87 | 182.10 | 43.67 | 71.45 |
| **G28** | 93.85 | 98.22 | 4.31 | 138.04 | 216.79 | 15.86 | 14.31 | 12.00 | 300.14 | 31.52 | 416.38 | 236.25 | 160.57 | 38.56 | 67.97 |
| **G29** | 91.57 | 96.05 | 4.41 | 135.05 | 212.82 | 15.58 | 14.39 | 13.86 | 457.54 | 32.52 | 603.08 | 280.01 | 189.66 | 31.45 | 67.73 |
| **G30** | 98.37 | 101.62 | 3.11 | 138.61 | 210.14 | 15.80 | 14.59 | 14.00 | 358.74 | 37.04 | 584.68 | 251.61 | 170.51 | 29.16 | 67.77 |
| **G31** | 98.49 | 102.49 | 3.76 | 134.94 | 220.42 | 13.08 | 14.03 | 13.21 | 265.90 | 34.60 | 322.77 | 178.50 | 107.60 | 33.34 | 60.28 |
| **G32** | 98.87 | 101.99 | 2.95 | 139.41 | 217.72 | 15.50 | 14.58 | 14.13 | 486.92 | 34.19 | 394.16 | 241.67 | 149.71 | 37.98 | 61.95 |
| **G33** | 95.57 | 99.82 | 4.20 | 139.25 | 205.53 | 16.45 | 14.77 | 13.73 | 390.99 | 31.59 | 369.04 | 218.21 | 136.82 | 37.07 | 62.70 |
| **G34** | 96.71 | 101.09 | 4.31 | 140.92 | 210.95 | 16.88 | 13.20 | 12.47 | 329.80 | 34.19 | 479.35 | 229.17 | 116.84 | 24.38 | 50.99 |
| **G35** | 95.46 | 99.36 | 3.80 | 137.66 | 201.58 | 15.97 | 14.37 | 13.47 | 303.76 | 30.87 | 504.42 | 228.84 | 115.56 | 22.91 | 50.50 |
| **G36** | 105.19 | 109.55 | 4.31 | 142.37 | 179.60 | 13.30 | 14.10 | 14.16 | 306.58 | 23.62 | 335.89 | 205.73 | 89.05 | 26.51 | 43.28 |
| **G37** | 102.75 | 106.82 | 3.83 | 138.41 | 144.49 | 12.12 | 12.80 | 12.71 | 182.85 | 32.55 | 239.05 | 114.30 | 78.65 | 32.90 | 68.81 |
| **G38** | 96.10 | 99.73 | 3.56 | 134.10 | 180.57 | 15.36 | 11.82 | 11.43 | 334.04 | 22.00 | 272.54 | 129.10 | 98.13 | 36.01 | 76.01 |
| **G39** | 99.57 | 103.84 | 4.20 | 137.77 | 176.42 | 14.77 | 12.01 | 12.43 | 378.72 | 23.82 | 254.96 | 146.79 | 106.96 | 41.95 | 72.86 |
| **G40** | 98.71 | 103.23 | 4.41 | 140.63 | 162.95 | 13.89 | 12.17 | 11.73 | 197.58 | 34.13 | 327.93 | 150.77 | 106.99 | 32.62 | 70.96 |
| **G41** | 91.72 | 96.27 | 4.36 | 135.47 | 160.52 | 13.94 | 12.41 | 12.00 | 242.60 | 35.55 | 377.11 | 172.15 | 117.36 | 31.12 | 68.17 |
| **G42** | 102.52 | 106.75 | 4.16 | 136.55 | 132.75 | 12.80 | 13.17 | 11.87 | 241.58 | 19.51 | 185.15 | 101.59 | 66.98 | 36.17 | 65.93 |
| **G43** | 98.72 | 103.27 | 4.36 | 138.57 | 136.12 | 17.61 | 11.63 | 11.96 | 258.78 | 32.53 | 315.38 | 147.41 | 99.18 | 31.45 | 67.28 |
| **G44** | 97.67 | 101.77 | 3.88 | 133.60 | 140.94 | 14.19 | 12.15 | 12.00 | 254.29 | 22.76 | 380.37 | 170.70 | 106.10 | 27.89 | 62.15 |
| **G45** | 100.82 | 104.18 | 3.27 | 139.18 | 168.99 | 16.34 | 12.51 | 11.21 | 325.98 | 36.34 | 632.95 | 322.81 | 169.09 | 26.71 | 52.38 |
| **G46** | 99.54 | 103.66 | 3.99 | 131.28 | 188.59 | 17.26 | 13.76 | 12.00 | 321.66 | 27.36 | 373.52 | 212.75 | 131.68 | 35.25 | 61.90 |
| **G47** | 89.76 | 93.70 | 3.83 | 127.62 | 162.91 | 14.51 | 10.93 | 11.96 | 170.48 | 16.03 | 212.91 | 120.93 | 78.27 | 36.76 | 64.72 |
| **G48** | 92.99 | 97.38 | 4.14 | 128.27 | 146.65 | 12.77 | 11.27 | 11.69 | 201.55 | 23.46 | 247.05 | 173.92 | 104.33 | 42.23 | 59.99 |
| **G49** | 92.29 | 96.15 | 3.80 | 130.65 | 160.54 | 15.03 | 11.99 | 13.47 | 331.40 | 14.27 | 303.96 | 155.10 | 108.54 | 35.71 | 69.98 |
| **G50** | 102.60 | 107.10 | 4.47 | 134.71 | 155.22 | 15.16 | 11.92 | 10.95 | 194.08 | 24.08 | 442.97 | 184.19 | 110.49 | 24.94 | 59.99 |
| **G51** | 104.98 | 109.04 | 3.94 | 139.25 | 147.55 | 12.78 | 11.75 | 12.38 | 270.41 | 19.31 | 340.64 | 158.59 | 96.92 | 28.45 | 61.11 |
| **G52** | 103.62 | 107.31 | 3.51 | 137.77 | 132.85 | 12.38 | 11.72 | 12.23 | 228.70 | 25.14 | 295.31 | 150.74 | 85.87 | 29.08 | 56.96 |
| **G53** | 97.88 | 101.48 | 3.44 | 136.10 | 198.19 | 13.13 | 12.47 | 11.21 | 291.56 | 23.49 | 418.30 | 210.39 | 97.22 | 23.24 | 46.21 |
| **G54** | 92.79 | 96.34 | 3.39 | 132.17 | 196.43 | 15.75 | 12.90 | 11.73 | 313.86 | 24.93 | 496.41 | 177.32 | 99.68 | 20.08 | 56.21 |
| **G55** | 94.22 | 98.88 | 4.54 | 140.06 | 187.69 | 10.95 | 12.09 | 11.47 | 195.10 | 23.46 | 304.98 | 172.47 | 99.74 | 32.70 | 57.83 |
| **G56** | 102.53 | 107.27 | 4.44 | 136.14 | 162.96 | 11.51 | 12.83 | 12.00 | 273.64 | 23.61 | 310.06 | 180.48 | 114.52 | 36.94 | 63.45 |
| **G57** | 100.69 | 105.09 | 4.11 | 134.83 | 166.64 | 11.90 | 10.92 | 11.47 | 262.39 | 23.27 | 295.10 | 155.10 | 88.70 | 30.06 | 57.19 |
| **G58** | 95.02 | 99.84 | 4.64 | 134.18 | 129.46 | 12.67 | 10.11 | 12.10 | 216.62 | 25.05 | 203.89 | 143.74 | 76.21 | 37.38 | 53.02 |
| **G59** | 104.39 | 108.31 | 3.83 | 134.09 | 139.39 | 12.16 | 11.42 | 12.00 | 279.53 | 25.26 | 258.37 | 159.31 | 76.14 | 29.47 | 47.79 |
| **Mean** | **97.68** | **101.90** | **4.08** | **136.32** | **166.77** | **14.23** | **12.41** | **12.57** | **267.58** | **26.64** | **303.57** | **164.03** | **107.62** | **37.64** | **67.01** |
| **Max** | **105.19** | **109.55** | **5.17** | **142.37** | **220.42** | **18.25** | **14.8** | **14.68** | **486.92** | **37.04** | **632.95** | **322.81** | **203.17** | **60.12** | **81.69** |
| **Min** | **87.71** | **91.35** | **2.95** | **127.62** | **113.02** | **10.95** | **9.64** | **10.47** | **120.81** | **11.18** | **142.88** | **95.1** | **62.28** | **20.08** | **43.28** |

**Figure S1:** **Weather data for two consecutive seasons 2023 (summer and winter) and 2024 (summer and winter)**

Climatic data summer 2023

|  | Rain (mm) | Temp (min) | Temp (max) |
| --- | --- | --- | --- |
| feb | 0 | 7.2 | 16.3 |
| feb | 2.4 | 10.8 | 24.7 |
| feb | 3.8 | 10.7 | 26.2 |
| feb | 7.8 | 11.7 | 25.7 |
| mar | 0 | 9.6 | 23.8 |
| mar | 0 | 12.1 | 31 |
| mar | 0 | 13.2 | 30.2 |
| mar | 0 | 18 | 35 |
| apr | 0 | 17.5 | 36.7 |
| apr | 0 | 19.1 | 38.8 |
| apr | 0 | 21.9 | 39.5 |
| apr | 0 | 21.7 | 40.9 |
| apr | 0 | 18.7 | 33.5 |
| may | 0 | 23.2 | 36.3 |
| may | 0 | 24.7 | 40.6 |
| may | 0 | 27 | 40.3 |
| may | 0 | 28.5 | 42.8 |
| jun | 9.4 | 25.4 | 40.8 |
| jun | 0 | 28.5 | 43.6 |

Climatic data winter 2023

|  | Rain (mm) | Temp (min) | Temp (max) |
| --- | --- | --- | --- |
| Nov | 0 | 14.3 | 28.8 |
| Nov | 0 | 10.5 | 26.4 |
| Nov | 0 | 9.2 | 26.1 |
| Dec | 0 | 7.7 | 26.7 |
| Dec | 0 | 4 | 23.1 |
| Dec | 0 | 6.2 | 24.6 |
| Dec | 0 | 11 | 20.8 |
| Dec | 0 | 7.1 | 15.5 |
| Jan | 2 | 6.7 | 19.1 |
| Jan | 0 | 8.8 | 20.2 |
| Jan | 0 | 7 | 22.6 |
| Jan | 0 | 7.5 | 23.7 |
| feb | 0 | 7.6 | 25.3 |
| feb | 0 | 9.4 | 26.7 |
| feb | 0 | 10.7 | 27.1 |
| feb | 0 | 12.5 | 27.6 |
| Mar | 0 | 12 | 28.9 |
| Mar | 0 | 14.5 | 35 |
| Mar | 0 | 15 | 34.9 |
| Mar | 0 | 14.8 | 35.5 |
| Mar | 0 | 16.7 | 37.9 |
| Apr | 30.6 | 21.2 | 34.1 |
| Apr | 0 | 22.7 | 37.4 |
| Apr | 0 | 20.6 | 39.6 |

Climatic data winter 2024

|  | Rain (mm) | Temp (min) | Temp (max) |
| --- | --- | --- | --- |
| Oct | 0 | 20.5 | 30.6 |
| Oct | 30 | 19.2 | 27.6 |
| Nov | 0 | 15.2 | 29.4 |
| Nov | 0 | 9.8 | 27.5 |
| Nov | 0 | 11.4 | 26.7 |
| Nov | 0 | 9.5 | 26.3 |
| Dec | 0 | 6.7 | 24.5 |
| Dec | 0 | 7.9 | 24.3 |
| Dec | 0 | 8.4 | 16.1 |
| Dec | 0 | 10.7 | 18.5 |
| Dec | 0 | 10.2 | 17.5 |
| Jan | 0 | 7.1 | 17.2 |
| Jan | 0 | 5.2 | 19.7 |
| Jan | 0 | 6.1 | 22 |
| Jan | 4 | 9.7 | 24 |
| Feb | 5.2 | 10.5 | 20.9 |
| Feb | 0 | 8.8 | 23.8 |
| Feb | 0 | 9.8 | 26.1 |
| Feb | 0 | 12.5 | 27.7 |
| Mar | 0 | 11.6 | 30 |
| Mar | 0 | 16.6 | 32.1 |
| Mar | 0 | 17.1 | 34.6 |
| Mar | 10.8 | 15 | 32.2 |

Climatic data summer 2024

|  | Rain (mm) | Temp (min) | Temp (max) |
| --- | --- | --- | --- |
| feb | 0 | 7.6 | 25.3 |
| feb | 0 | 9.4 | 26.7 |
| feb | 0 | 10.7 | 27.1 |
| feb | 0 | 12.5 | 27.6 |
| Mar | 0 | 12 | 28.9 |
| Mar | 0 | 14.5 | 35 |
| Mar | 0 | 15 | 34.9 |
| Mar | 0 | 14.8 | 35.5 |
| Mar | 0 | 16.7 | 37.9 |
| Apr | 30.6 | 21.2 | 34.1 |
| Apr | 0 | 22.7 | 37.4 |
| Apr | 0 | 20.6 | 39.6 |
| Apr | 0 | 24.4 | 35.7 |
| May | 16.6 | 25.2 | 38.2 |
| May | 0 | 25.5 | 39.1 |
| May | 57.4 | 24.5 | 35.8 |
| May | 18.8 | 26.8 | 37.8 |
| Jun | 0 | 26 | 38.2 |
| Jun | 0 | 28.4 | 39.5 |
| Jun | 55.6 | 26 | 33.9 |

**Supplementary File S1. Statistical Analysis Workflow and Software Information**

Software Environment

All analyses were performed in R version 4.5.1 (R Core Team, 2025).

Packages Used

• metan (v1.19.0) – mixed-model analysis, BLUP estimation, WAASB/WAASBY, MGIDI, MTSI, MTMPS, and FAI-BLUP analyses.

• sommer – mixed-model and factor analytic (FA1) covariance structure modelling.

• dplyr and tidyr – data processing and manipulation.

• ggplot2 – graphical visualization.

• GGally – trait association matrix plots.

• circlize – chord diagram visualization.

• ggvenn – overlap and coincidence analyses.

Analytical Workflow

1. Multi-environment trial data were analysed using mixed models.
2. Variance components, heritability, and BLUPs were estimated.
3. Stability analyses were conducted using WAASB and WAASBY.
4. Multi-trait genotype evaluation was performed using MGIDI, MTSI, MTMPS, and FAI-BLUP indices.
5. Coincidence analysis was used to identify consensus superior genotypes across selection methods.
6. Factor analytic (FA1) covariance structures were fitted to evaluate genotype × environment interactions.
7. Graphical outputs were generated using the packages listed above.
